# Petri Net computational modelling of Langerhans cell Interferon Regulatory Factor Network predicts their role in T cell activation

**DOI:** 10.1038/s41598-017-00651-5

**Published:** 2017-04-06

**Authors:** Marta E. Polak, Chuin Ying Ung, Joanna Masapust, Tom C. Freeman, Michael R. Ardern-Jones

**Affiliations:** 1 Clinical and Experimental Sciences, Sir Henry Wellcome Laboratories, Faculty of Medicine, University of Southampton, SO16 6YD Southampton, UK; 2 Institute for Life Sciences, University of Southampton, SO17 1BJ Southampton, UK; 3 The Roslin Institute and Royal (Dick) School of Veterinary Studies, University of Edinburgh, Easter Bush, Edinburgh, Midlothian EH25 9RG UK

**Keywords:** Gene regulation in immune cells, Langerhans cells

## Abstract

Langerhans cells (LCs) are able to orchestrate adaptive immune responses in the skin by interpreting the microenvironmental context in which they encounter foreign substances, but the regulatory basis for this has not been established. Utilising systems immunology approaches combining *in silico* modelling of a reconstructed gene regulatory network (GRN) with *in vitro* validation of the predictions, we sought to determine the mechanisms of regulation of immune responses in human primary LCs. The key role of Interferon regulatory factors (IRFs) as controllers of the human Langerhans cell response to epidermal cytokines was revealed by whole transcriptome analysis. Applying Boolean logic we assembled a Petri net-based model of the IRF-GRN which provides molecular pathway predictions for the induction of different transcriptional programmes in LCs. *In silico* simulations performed after model parameterisation with transcription factor expression values predicted that human LC activation of antigen-specific CD8 T cells would be differentially regulated by epidermal cytokine induction of specific IRF-controlled pathways. This was confirmed by *in vitro* measurement of IFN-γ production by activated T cells. As a proof of concept, this approach shows that stochastic modelling of a specific immune networks renders transcriptome data valuable for the prediction of functional outcomes of immune responses.

## Introduction

In order for the immune system to provide effective defence against pathogens and xenobiotics, it is critically important that it discriminates between signals that indicate danger and those which are non-threatening and to which a “passive” or “tolerant” response is appropriate. Modulation of immune regulation is of particular importance at body surfaces such as skin, where programming the adaptive immune responses takes place^1^. Here a CD1a high, CD207+ subset of cutaneous dendritic cells, Langerhans’ cells (LCs), initiate a rapid immune response to an inflammatory signal from the tissue environment^2, 3^. However, in steady state conditions, LCs selectively induce the activation and proliferation of skin-resident regulatory T cells^4, 5^ that help prevent unwanted immune-mediated reactions.

This important balance is impaired in inflammatory skin conditions such as atopic dermatitis (AD), where disseminated herpes simplex virus (HSV) infection can be life-threatening without effective treatment^6^. Recently a number of risk factors which may predispose patients with AD to develop eczema herpeticum have been identified, including filaggrin mutations, high serum IgE levels and reduced levels of IFN type I and II^7–9^. However, the molecular mechanism underpinning the susceptibility to herpes virus infection remains poorly understood. Aberrations observed in eczema herpeticum patients point to the importance of impaired anti-viral immune response^10^, diminished activation of CD8+ cytotoxic T cells^11^, and production of indoleamine 2,3-dioxygenase by antigen presenting cells residing in the skin^12^. We and others have shown, that LCs play a central role in the regulation of CD8 T cell-mediated cytotoxic immunity through their unique ability to efficiently cross-present antigens and induce effective CD8 T cell responses^2, 3, 13^. In atopic disease the ability of skin dendritic cells to polarise adaptive immune responses towards Th2 and Th22 through the effect of aberrant cytokine signalling has been documented in previous studies^1, 14–16^. However, little is known of how this signalling affects the ability of LCs to induce CD8 T cell function.

A growing body of evidence suggests that the decision processes which control immune activation or tolerance are executed via simultaneous signalling through multiple transcription factors interconnected in complex molecular networks^17, 18^. In particular, immune regulation at the transcriptomic level seems to be executed via gene regulatory networks (GRN). These provide causal molecular explanations for cellular behaviour and execution of transcriptomic programmes, as they detail in a directed manner the flow of genomic information and the control of cellular outputs^19–21^.

The ability to comprehensively analyse signalling events in LC GRN is essential for understanding of immune regulation in human skin. While it is relatively easy to manipulate the stimulus properties and environmental conditions *in vitro*, the comprehensive assessment of the signalling dynamics in intact human skin is beyond the limits of experimental science. Computational modelling offers the most promising way to approach the problem, providing the mathematical framework for modelling the resting state of signalling systems, including disease-specific steady states, predicting the cell and system behaviour during prolonged exposure to signalling stimulus, and the outcome of multiple signalling events^19, 22^.

Quantitative models, using Michaelis-Menten kinetics-based rate laws^23^ and mass action kinetic models^20^, have been successfully used for simulating small biological networks. They have provided insights into the mechanisms regulating gene, signalling and metabolic regulatory network behaviour. However, the inherent limitation of such an approach lies with the requirement for input of detailed kinetic parameters and relationships within the network, hence constraining the models to relatively small sized networks. For analysis and modelling of large molecular networks, including metabolic networks^21^, signalling networks^24^ and gene regulatory networks^25^, Petri nets have recently emerged as a promising tool. The approach allows the user to vary inputs, which then create a signal flow through the network based solely on the network connectivity, eliminating the necessity for multiple kinetic parameters at each step. The network model was first validated to recapitulate outcomes reported in the literature, including dendritic cells and macrophage subsets^26–35^. Subsequently they have been used to model experimental data derived from whole transcriptome analysis of human Langerhans cells.

To understand better the molecular cross-talk between the structural cells and LC orchestrating adaptive immune responses, we have applied a bioinformatic analysis of transcriptomics data. This allows network inference and dynamic simulation of the behaviour of transcription factor networks and experimental validation of model predictions. Combining bioinformatics analysis with *in vitro* experiments has allowed us to characterise the differential effect of key epidermal cytokines, TNFα and TSLP, on the ability of LCs to cross-present viral antigens to cytotoxic T cells, and to propose a transcriptional mechanism regulating this process.

## Results

### Epidermal cytokines, TNFα and TSLP, differentially regulate the expression of Interferon Regulatory Factors in human migratory LCs

**Figure 1:**
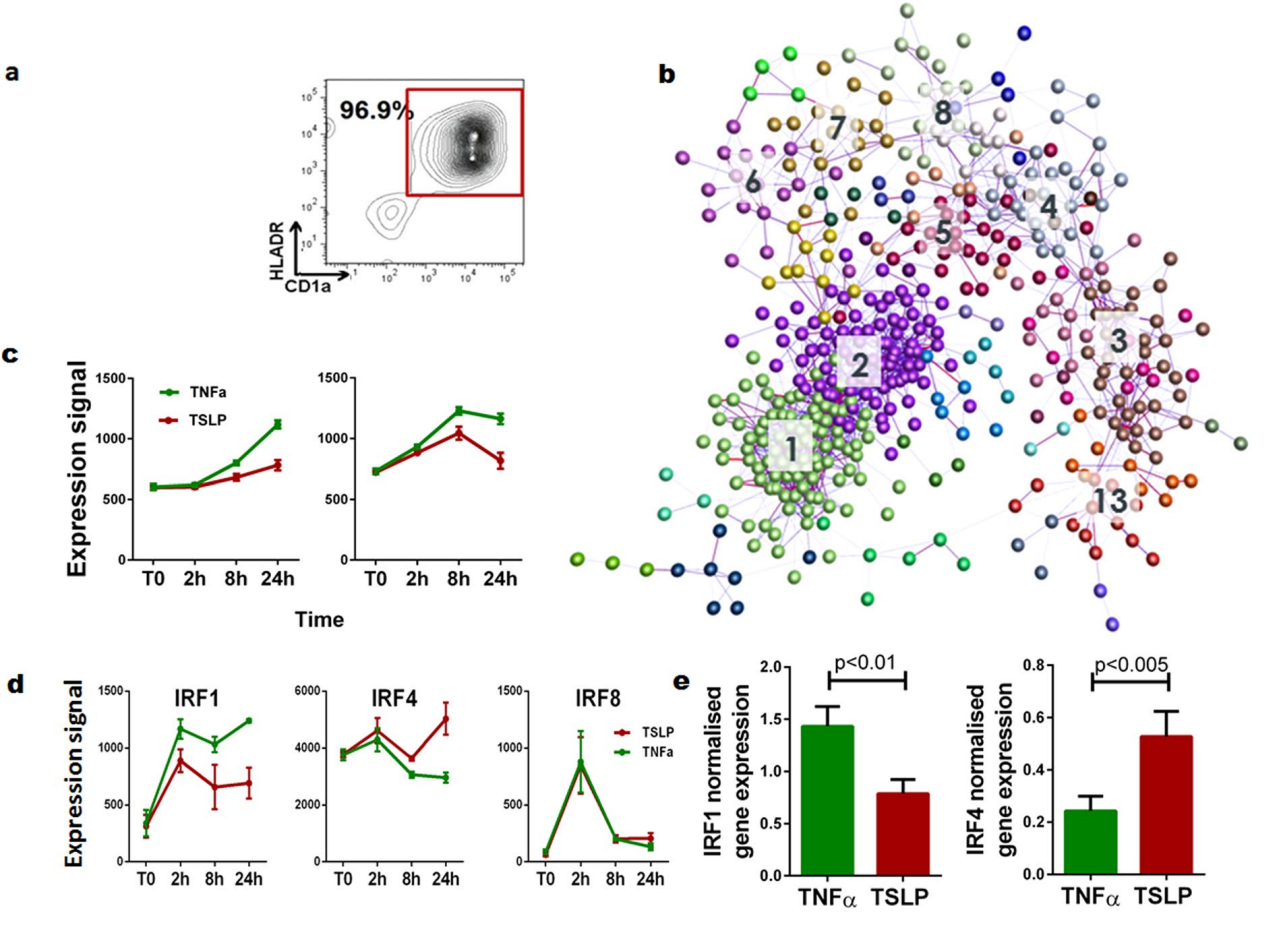
Changes in Langerhans cell core transcriptional network induced by epidermal cytokines are associated with a dramatic change in expression of IRF1, 4, and 8. (**a**) Freshly isolated 48 h migratory human LCs are CD1a/HLADR^high^. (**b**) The core transcriptomic networks of human LCs comprising 17 clusters, including 2 biggest clusters (01 and 02) of genes involved in antigen processing. Transcript-to-transcript clustering, (BioLayout *Express* ^3D^, r = 0.85; MCL = 1.7) of 527 probesets differentially regulated during 24 h of stimulation with TNFα and TSLP, maSigPro p < 0.05. Lines (edges) represent the similarity between transcript expressions; circles (nodes) represent transcripts. Clusters of co-expressed genes are coded by colour. (**c**) Expression profile of clusters 01 (95 genes) and 02 (85 genes) during 24 h stimulation with epidermal cytokines, green: TNFα, red: TSLP). (**d**) Expression changes of *IRF1*, *IRF4* and *IRF8* in LC during the time course of stimulation with TNFα and TSLP, n = 3 independent skin donors. (**e**) Differential induction of *IRF1* and *IRF4* mRNA by TNFα and TSLP during LC migration from biopsies (qPCR, cells from four 6 mm skin biopsies, n = 6 in duplicate, mean ± SEM, p < 0.0001 for IRF1 and IRF8, and 0.013 for IRF4, two-way repeated measurements paired ANOVA).

Our recent study documented that TNFα-matured LCs express a characteristic molecular signature comprising genes involved in antigen capture, intracellular trafficking and formation of immunoproteasome, rendering them superior activators of anti-viral CD8 T cell responses^2^. To analyse how this molecular signature is regulated by signalling from atopic keratinocytes, we measured the whole transcriptome expression of the human migratory LCs (85–96% CD1a+/HLA−DR+ (Fig. 1a)) during a time course stimulation with TSLP. Bayesian Estimation of Temporal Regulation (BETR)^36^ identified 870 probesets up-regulated at 2 h, 349 up-regulated at 8 h and 280 up-regulated at 24 h of stimulation with TSLP in comparison to unstimulated migratory cells. Following exposure to TNFα, probesets up-regulated were 789, 524, and 482 at the corresponding time points. TSLP induced down-regulation of 118 probesets (2 h), 618 probesets (8 h) and 613 probesets (24 h) (compared to 302, 895, and 772 probesets down-regulated by TNFα at the corresponding time points, 1 fold difference in log 2(x) robust multichip average (RMA)-normalized expression level between the time point and control, BETR p < 0.05). Comparative analysis of whole transcriptome data from human LCs matured with TNFα or TSLP defined a core signature of 527 genes as being differentially regulated (maSigPro algorithm^37^, p < 0.05) by the two cytokines (Fig. 1b). A transcript-to-transcript Pearson correlation matrix was calculated, a graph constructed in BioLayout *Express* ^3D^ ^38^ (*r* = >0.8) and subjected to clustering using Markov Clustering Algorithm (MCL)^39^ with an inflation value set at 1.7 (this controls the granularity of clustering) and the smallest cluster size set at 5. The analysis identified 18 clusters, 5 preferentially up-regulated by TNFα. The two largest clusters (01 and 02) of genes are involved in induction of immune responses and underpinning the cellular processing of antigens (Fig. 1b). They included genes encoding proteins involved in antigen capture (*CAV1*), intracellular trafficking (*SNX10* and *SNX11*) and formation of immunoproteasome (*PSME2*, *PSME3*, *PSMB10*), (Fig. 1c). The 13 smaller clusters induced preferentially by TSLP included genes involved in kinase signalling, peroxisome function and nucleotide metabolism. The full details of gene ontology enrichment of the identified clusters are listed in Table 1, p values calculated using 2 way repeated measurement paired ANOVA, for time and cytokine variable.

**Table 1:** Gene Ontology enrichment in clusters preferentially induced by TNFα or TSLP signalling.

Cluster	Preferentially regulated by (time, cytokine, two way ANOVA)	gene number	GO (FDR B&H)/gene list for low gene number clusters
01	TNFα (p < 0.0001, p = 0.021)	95	immune response (p = 0.0051), leukocyte activation (p = 0.0051), proteasome activator complex (p = 0.009)
02	TNFα (p < 0.0001, p = 0.011)	84	Pathways: cell cycle (p = 0.008), HIV infection (p = 0.012), proteasome (p = 0.036), cross-presentation of soluble exogenous antigens (endosomes) (p = 0.036),
09	TNFα (p < 0.0001, p = 0.052)	12	regulation of RNA splicing (p = 0.015)
17	TNFα (p < 0.0001, p = 0.018)	6	*CLIP2*, *IL1R2*, *OAF*, *RAB38*, *TCF7*, *TMEM184C*
18	TNFα (p = 0.0002, p = 0.002)	6	*C17orf62*, *C19orf54*, *CPNE1*, *FTSJD2*, *HECW1*, *STK25*
03	TSLP (p < 0.0001, p = 0.005)	36	no annotation
04	TSLP (p = 0.006, p = 0.001)	25	no annotation
05	TSLP (p < 0.0001, p = 0.019)	25	JUN kinase binding (p = 0.027)
06	TSLP (p < 0.0001, p = 0.001)	18	peroxisome proliferator activated receptor binding (p = 0.017)
07	TSLP (p < 0.0001, p = 0.004)	18	no annotation
08	TSLP (p < 0.0001, p = 0.007)	16	nucleotide transferase activity (p = 0.026)
10	TSLP (p < 0.0001, p = ns)	10	nucleotide metabolism (p = 0.042)
11	TSLP (p < 0.0001, p = 0.022)	10	mRNA splicing (p = 0.025)
12	TSLP (p = 0.0007, p = 0.021)	10	Golgi aparatus (p = 0.025)
13	TSLP (p < 0.0001, p = 0.038)	9	transferrin receptor activity ((p = 0.001)
14	TSLP (p = 0.0014, p = 0.003)	8	*ATP5L*, *EFHA1*, *ID2*, *INIP*, *RECQL*, *RPS4X*, *TMSB4X*, *UBL5*
15	TSLP (p < 0.0001, p = 0.054)	8	*CAMK1D*, *ELL3*, *LAP3*, *MLLT4*, *MPC1*, *NET1*, *NFE2L3*, *STOM*
16	TSLP (p < 0.0001, p = ns)	7	*ARAP1*, *CNDP2*, *GSDMD*, *N4BP2L2*, *NINJ2*, *PARP10*, *VPS13B*

To better understand the gene regulatory networks we analysed the expression modules of co-expressed LC genes, and the identified promoter motifs and corresponding transcription factors (TF) which regulate their expression. In LCs treated with the two cytokines the differentially regulated genes contained a possible IRF binding site STTTCRNTTT as the main binding site enriched in the gene signature (ToppGene^40^, BH p = 0.0125). Analysis of the expression of transcription factors indicated that at T_0_ *ZFP36L1*, *NFKBIA*, and *NFKB1* were the most highly expressed transcription factors. However, following stimulation of LC with the inflammatory cytokine TNFα, there was dramatic up-regulation of *IRF1* and *IRF8* transcripts (6.7 and 13.5 fold respectively) at the earliest time point, BETR, p < 0.001) (Fig. 1d). In agreement with their potential role as mediators of LC responses to TNFα and TSLP, the top differentially regulated TFs were *IRF1*, *IRF4* and *IRF8*, and IRF transcriptional partners including *JUN*, *ATF3*, *BATF*, *BATF3;* (assessed both from absolute expression levels and fold change difference (Fig. 1d and Supplementary Figure S1)). The dependence of *IRF1* and *IRF4* expression levels on the cytokines present in the tissue microenvironment was further confirmed in LCs migrating from the epidermal biopsies exposed to TNFα or TSLP (Fig. 1e).

### Stochastic simulation of a logic-based diagram of the IRF gene regulatory network with Petri Nets allows recapitulation of dendritic cell- induced T cell polarisation

To regulate the cellular functional outcome, IRFs interact in a synergistic or antagonistic manner with other transcription factors and adaptor molecules^41–44^. We hypothesised that these interactions create a gene regulatory network encoding the transcriptional programmes in dendritic cells. To address the complexity of the interactions within this GRN, we assembled a logic-based diagram to capture the multiple reported interactions between IRFs, IRF transcription partners and DNA sequences, orchestrating gene transcription, cell function, and thus, the outcome of immune stimulation^45, 46^ (Supplementary Materials and Methods, Tables S1–S4, Supplementary Figure S2). To identify components for the IRF-GRN, a systematic search was performed in PubMed for terms describing involvement of IRFs in dendritic cell function, antigen presentation and T cell function (Supplementary Table S1). From the 618 returned papers, 83 unique original papers were identified, describing regulation of gene expression by IRF and their transcription partners (Table S2). The data has been structured into an interaction database containing entries for: (1) and (2) interaction partners, (3) mode of interaction, (4) DNA sequence, (5) regulated genes, (6) biological process. This was subsequently converted into a matrix of Boolean interactions between the network components (Table S3) and computationally modelled using a version of Stochastic Petri Nets (SPN)^46^. For a detailed description of a diagram assembly please refer to Livigni *et al*.^45^ and the methods section of this manuscript^45^.

**Figure 2:**
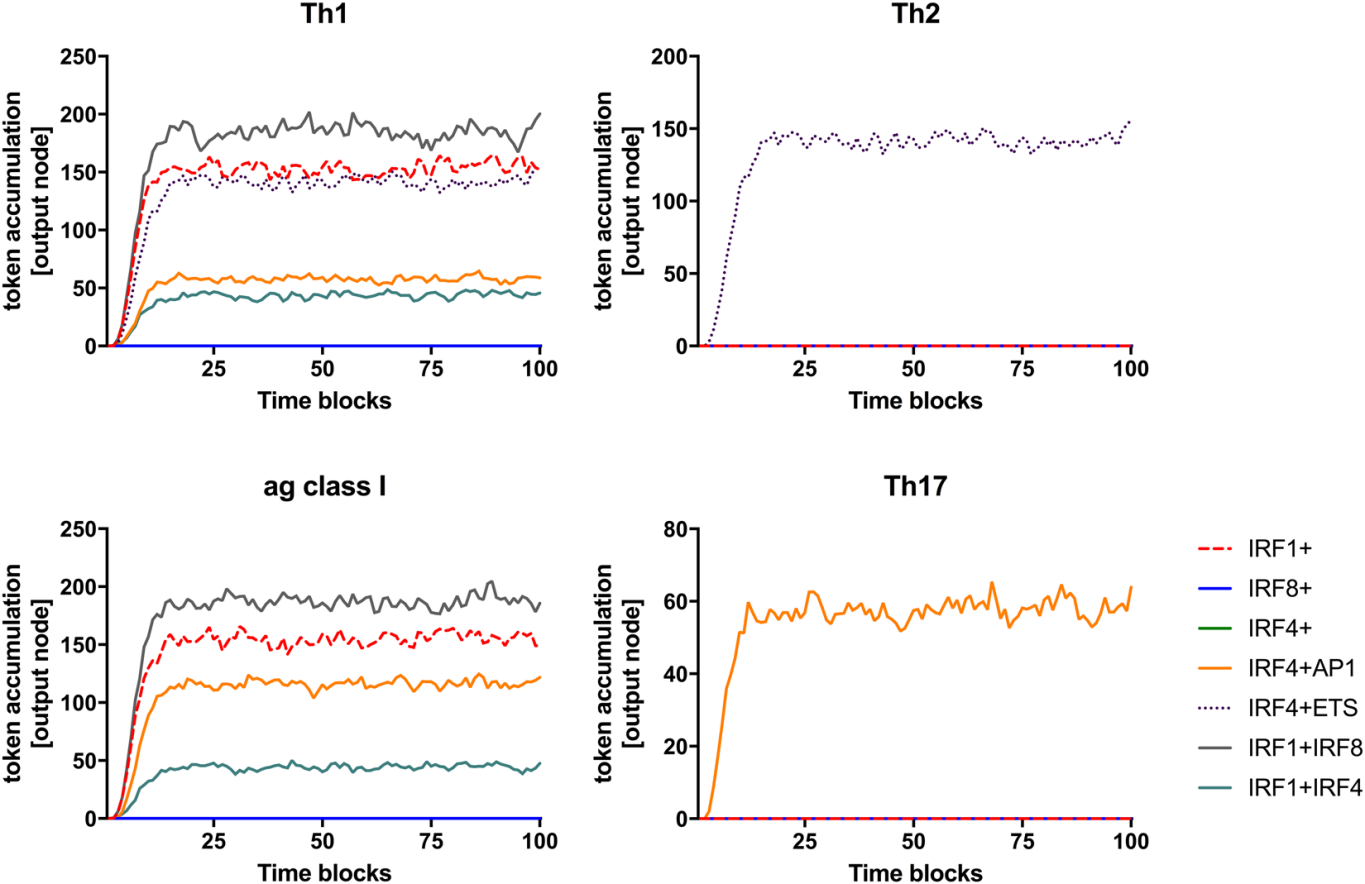
Network of IRF and their transcription partners regulates transcriptional programmes of dendritic cells. Model of IRF-GRN assembled based on a systematic literature review have been simulated with Signalling Petri Nets in BioLayout *Express* ^3D^. Representative results of in silico simulation of the IRF network, measured at each of the output nodes, when *IRF1* only (dotted red), *IRF8* (blue), *IRF4* only (green), *IRF4* and AP1-binding TF (orange), *IRF4* and ETS-binding TF (dotted purple), *IRF1* and *IRF8* (grey) and *IRF1* and *IRF4* (turquoise) are expressed.

The initial validation of the IRF-GRN was performed using theoretical quantities, where “0” represented lack of transcription factor expression, corresponding to a gene knock/out model. *In silico* simulation using the SPN algorithm (initial marking set up as a theoretical value either 0 (null expression) or 100 (expressed gene) for all possible combinations of the entry nodes: IRF1, IRF4, IRF8, AP1-binding and ETS-binding) demonstrated that the model correctly re-capitulates the observations from multiple experimental systems (Table S2, Fig. 2 and Figure S2). The data describe the involvement of IRFs and their transcriptional partners in regulation of a “stereotypical” antigen presenting cell function of a dendritic cell/macrophage lineage. As shown in Fig. 2b, all the conditions outlined have been met by the *in silico* model of the GRN. As reported by others, induction of Th1 responses required expression of either IRF1 alone or a combination of IRF8 and ETS transcriptional partners^26–28, 33^ (Fig. 2a, Table S2), while expression of Th2/Th17 was critically dependent on simultaneous expression of IRF4:ETS^29–31^ (Th2, Fig. 2b, Table S2) or IRF4:AP-1^32, 47^ (Th17 Fig. 2d, Table S2) binding partners. Likewise, the simulation recapitulated a cooperative involvement of IRFs and their transcription partners for induction of antigen presentation by MHC class I molecules, resulting in activation of CD8 T lymphocytes (in agreement with data from existing literature, Tables S2 and S3)^33, 34, 42^. It predicted that optimal induction of CD8 T cell activation requires signalling via both IRF1 and IRF8 (Fig. 2c)^35^.

### The modulation by epidermal cytokines of LC ability to activate antigen-specific CD8 T cell responses is predicted by *in silico* modelling of IRF-GRN parametrised with experimental data

One of the more surprising findings emerging from the initial studies of networks and component interactions in different cell types is the multi-functionality (‘functional pleiotropism’) of signalling networks. This suggests that biological networks have evolved to enable passing of biologically distinct information through shared channels^48^. In essence, while the GRN architecture and main components are shared between different cell types, the spectrum of output genes regulated by the network varies with the specific cell type. This can be illustrated by the fact that although, for Th1-polarising dendritic cells, IRF1-controlled IL12p70 production appears established^26, 49^, we and others have demonstrated that IL12p70 is not produced by human LCs^2, 13, 50, 51^. This is despite the rapid up-regulation of *IRF1* transcripts in LCs upon stimulation (Fig. 1d). The presented IRF-GRN has been assembled by us based on published data derived from multiple cell types. Therefore, to model the IRF-dependent programming within LC we adapted the generic IRF-GRN to represent the interactions reported in human primary LCs.

**Figure 3:**
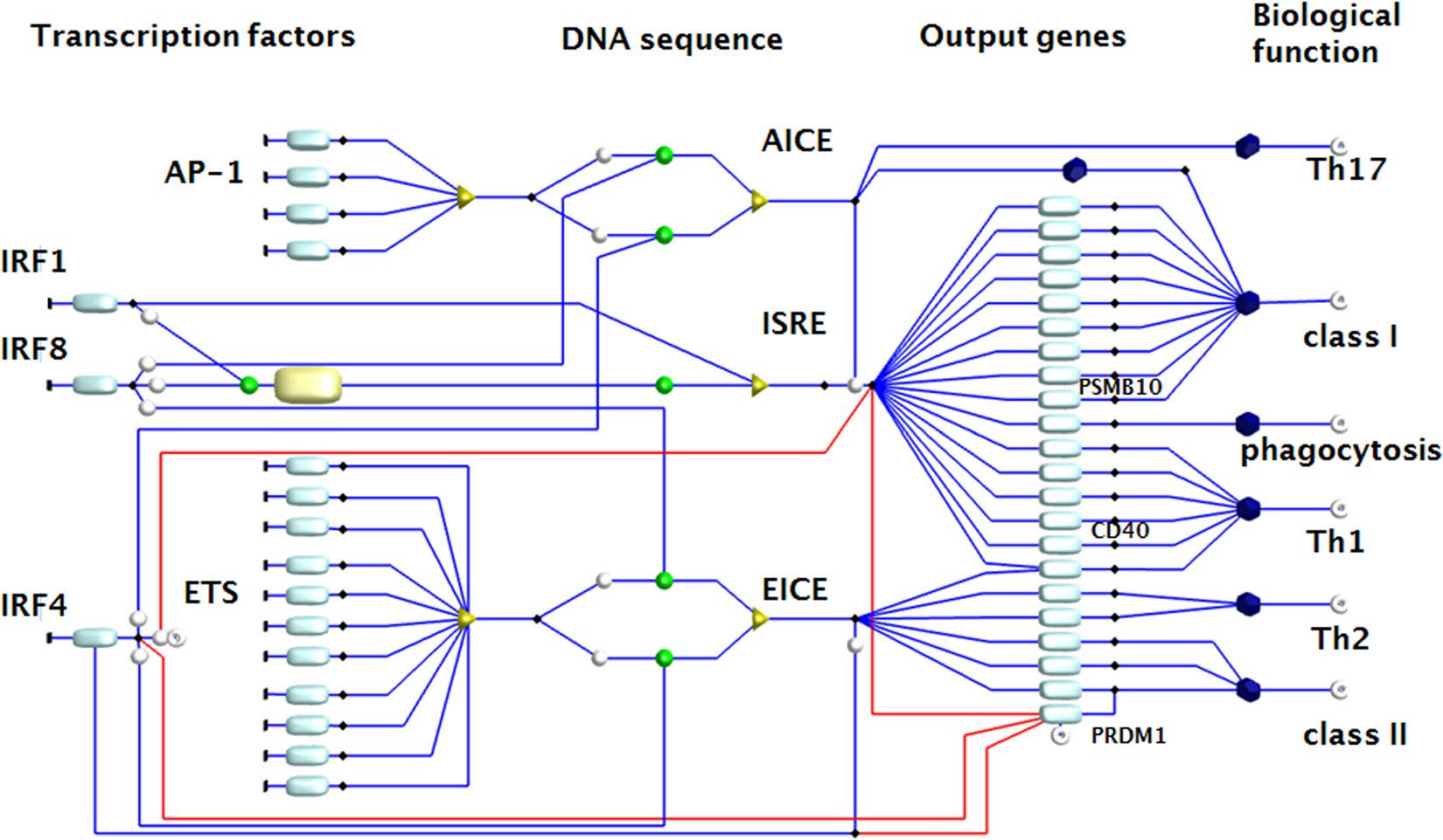
Network of IRF and their transcription partners underpins biological function of human Langerhans cells. Interferon Regulatory Factors gene regulatory network (GRN) in DCs, assembled basing on the systematic literature review, depicting *IRFs*, transcription partners, DNA sequences and transcribed genes arranged in columns from left to right. Components of the GRN are represented by rectangles (gene transcripts) and triangles (DNA sequences) connected by arrows representing molecular interactions (blue arrow: synergism, red arrow: inhibition). Green circle denotes binding. GRN output (i.e. immunological function) is presented in octagons on the right side of the diagram. The diagram is drawn in a Petri Net notation, where the interacting elements of GRN (nodes, gene transcripts) are interspaced with transitions (vertical black lines, and black diamonds). Input nodes: *IRF* 1, 4, and 8, and transcription partners grouped as ETS or AP-1 family. Assumption: IRF can bind with any TP from the ETS family. There are 28 members of ETS family, and 5 AP-1 binding transcription factors. Only the transcription partners exceeding 150 RMA normalised expression level in the human skin LC microarray dataset were included in the diagram. The nodes include (classes: left to right, list: top to bottom: Transcription factors: *IRF1*, *IRF8*, *IRF4*, IRF-binding partners: AP-1 family: *JUN*, *FOS*, *BATF*, *BATF3*, ETS family: *ELF1*, *ELF4*, *ELK1*, *ELK3*, *ETS1*, *ETS2*, *EHF*, *ELF2*, *ETV3*, *ETV6*, *GABPA*. DNA binding sequences: AICE, ISRE, EICE. Output genes: Programme A (bracket indicates output genes depicted in a single node): *CAV1*, *ERAP1*,*2*, *TAP1*, (*HLA A*-*F*, *B2M*), *TAP2*, *TAPBPL*, *PSME1*, *PSME2*, *PSMB10*, *CYBB*, (*CD40*, *CD80*, *CD86*), *IL15*, *IL12p40*, *IFNb*, *iNOS*, *IL18*. Programme B: *IL10*, *IL33*, *CD74*, *LYZ*, *CIITA*, *PRDM1*. Biological processes: Th17 responses, antigen presentation in class I, phagocytosis, Th1 responses, Th2 responses, antigen presentation in class II. Each interaction has been confirmed by two independent reports in myeloid cells. The diagram captures the combinatory nature of immune activation, depending on the levels of expression, timing and interactions between the regulatory elements. The flow of the signal through the diagram can be modelled mathematically using experimental or theoretical data and visualised in BioLayout Express^3D^. Programmes A (green) and B (red) are controlled by combinatorial binding of IRF-TP to different DNA sequences. The detailed diagram can be edited/downloaded from http://www.virtuallyimmune.org/irf-grn/.

**Figure 4:**
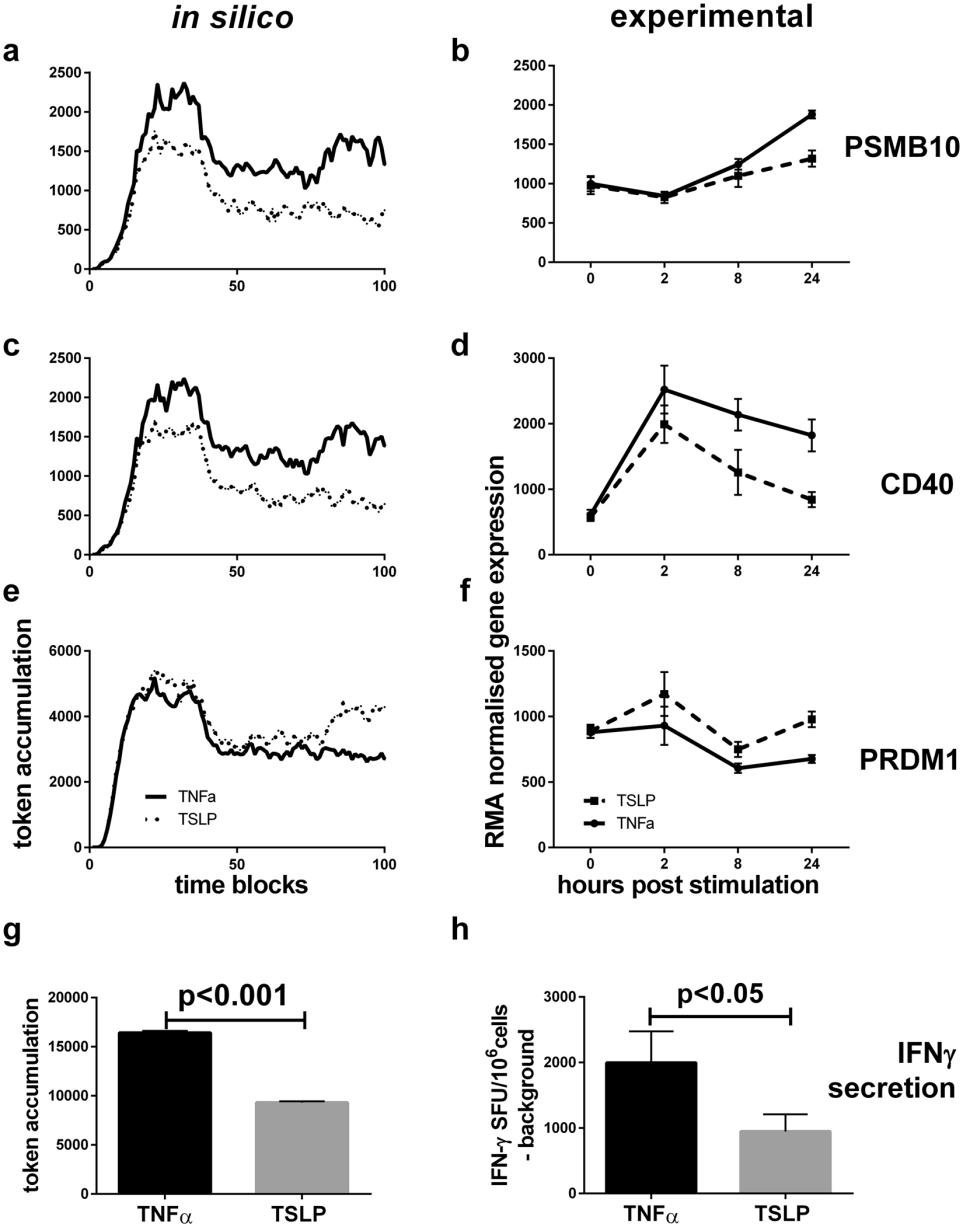
*In silico s*imulation of GRN predicts changes in expression of genes regulated by IRFs and the outcome of T lymphocyte stimulation by LCs. (**a**–**f**) Expression levels of *PSMB10* (**a**,**b**) *CD40* (**c**,**d**) and *PRDM1* (**e**,**f**) predicted in silico (**a**,**c**,**e**) and measured 24 h post *in vitro* activation of LCs (**b**,**d**,**f**). (**g**,**h**) The ability of TNFα (black) and TSLP (grey) matured LCs to stimulate antigen-specific CD8+ T cells was simulated *in silico* and measured in ELISpot *in vitro* assay. (**g**) Result of in silico simulation of the IRF network, measured at the output node when the input nodes are marked as per the gene expression values during LCs stimulation with TNFα and TSLP, Signalling Petri Nets: BioLayout *Express* ^3D^, 100 time blocks, 500 runs. Number of tokens in the output node in the 10 final time blocks shown. (**h**) Activation of antigen-specific CD8+ T cells by TNFα (grey) and TSLP (black) matured LCs, pulsed with a long peptide antigen requiring cross-presentation, IFNγ production measured in co-culture ELISpot assay, n = 6 in triplicate, mean+/−SE.

To test the ability of human LC to regulate adaptive immune responses, the GRN model was expanded to include all the members of AP-1 and ETS family found to be expressed in human LCs, determined by microarray analysis^2^ (Table S6). Furthermore, the network was enriched in elements representing output genes derived from existing IRF1, IRF4, and IRF8 ChiP-seq data (Table S4) and filtered to include only the genes expressed by human LCs, as measured by microarray experiments (GSE23618, GSE16395, GSE35340, GSE49475, Fig. 3). A series of values representing changing levels of LC gene expression (derived from the microarray data) over the time course of stimulation with TNFα or TSLP provided the initial values for all entry nodes (Table S6, Supplementary executable model files: http://www.virtuallyimmune.org/irf-grn/). Stochastic simulation of the flow of tokens through the network and the assessment of token accumulation in the network output nodes provided *in silico* predictions of the pattern of gene expression in LCs during the time course of stimulation by TNFα and TSLP. It also revealed their potential to induce different immune responses. Analysing the patterns of token accumulation at the network output nodes identified two distinct programmes of gene expression, “A” and “B”. Programme “A” included genes preferentially induced by TNFα after binding of transcription factors to ISRE and “B” comprised genes regulated in similar manner by TNFα and TSLP, induced after transcription factor binding to EICE (Figure S3). The results of simulation experiments correctly predicted whether the gene expression profile, as measured experimentally, belonged to programme “A” (genes up-regulated by TNFα) or programme “B” for 34 out of 50 of the network output genes. Predictions included genes associated with antigen presentation (*HLA*-*A*, -*B*, -*C*, *CIITA*, *HLA*-*DR*), immunoproteasome (*PSME1*, *PSME2*, *PSMB10*), LC activation (*CD40*), and endocytosis (*CAV1*) (Fig. 4a–f, Table S5, Supplementary Figure S3). Furthermore, simulation experiments indicated that the ability of LCs to present a peptide to CD8 T cells would be altered by the cytokine milieu (TNFα/TSLP), which has not previously been reported and was not anticipated. To test the *in silico* predictions, we have examined the ability of LC to cross-present antigens to antigen-specific CD8 T lymphocytes, utilising a long peptide (30 amino acid) containing the EBV BMLF-1 epitope. This epitope is restricted to HLA-A2 and requires intracellular processing for subsequent cross-presentation into the MHC Class I pathway^2, 3^. Consistent with the model prediction, maturation by TSLP diminished the capacity of LC to cross-present a viral epitope to antigen-specific CD8 T cells (Fig. 4g,h, n = 5, p < 0.05, Figure S4) whereas this was enhanced by TNFα.

Subsequently, to test if the model was capable of predicting LC behaviour when they have been exposed to signals or perturbations within intact epidermis, we targeted signalling of PI3Kγ. This kinase is highly expressed by LC, in contrast to dermal Dendritic Cells^2^ and is one of the most up-regulated genes induced by TSLP (Table 1, Cluster 05). *Ex vivo* epidermal biopsies were cultured in the presence or absence of AS605240, a potent, cell-permeable and ATP-competitive inhibitor of PI3Kγ^52^ (Figure S5a). Migratory LC (Figure S5b) were harvested 48 h later from inhibitor exposed and non-exposed biopsies and the LC transcriptome assessed using Affymetrix Human Gene ST 1.1 microarrays (n = 2 independent donors).

The *in silico* simulations, run using normalised transcription levels as the initial marking for IRF-GRN input nodes (Table S7), predicted that the ability of LC to induce activation of Th1 but not Th2 responses will be diminished by the inhibitor (Figure S5c,d). To validate the *in silico* predictions and assess the ability of LC to prime and polarise adaptive immune responses, LC were co-cultured with allogeneic naïve CD4 T cells for 6 days. Secretion of IFNγ and IL-4 by primed T cells was used as a proxy for Th1 and Th2 polarisation (Figure S5e,f, n = 6, in triplicate, mean ± SEM shown). As shown in Figure S5, *in vitro* validation confirmed that the PI3Kγ inhibitor reduced the LCs ability to induce Th1 immune responses (p < 0.05). The observed trend in reducing production IL-4 was not statistically significant, as predicted *in silico*.

## Discussion

Molecular targeting of key signals in the immune system has already demonstrated significant advances in the treatment of human disease including cancer and inflammation. These new therapies depend on targeting single molecules or pathways. To date, most such treatments have focused on known effector pathways in immunity such as T cell cytokines. However, yet undiscovered potential lies in targeting factors critical to maintenance of the aberrant immune responses, for which dendritic cells are likely to hold the key. Here, we proposed to investigate in detail the role of immune regulation at the level of transcriptomic networks in human LCs responding to cytokine signals, modelling inflamed epidermis. LCs’ anatomical location in the outermost part of the skin and mucosal tissue, combined with their classical capacity for antigen capture, processing and presentation, make a strong case for their role as the primary gatekeepers against infection and other exogenous pro-inflammatory stimuli. By focusing on a key control element of immune regulation by LCs, we have identified the molecular basis for the orchestration of epidermal immunity, which may potentially offer molecular targets for immune intervention.

Analysis of transcriptional networks allowed us to identify a set of transcription factors from the Interferon Regulatory Factors (IRF) family, as a key GRN operating in human LCs. IRFs are critical regulatory molecules for dendritic cell development and function^42, 53, 54^, as well as for efficient regulation of immune responses to infectious pathogens^26, 55, 56^. The importance of IRFs for tissue homeostasis has been further highlighted by the association of the causal disease variants in GWAS studies. Thus, IRF-binding sequences have been linked with autoimmunity and inflammatory skin disease^57, 58^, and their key role in driving the Th2 phenotype dominant in asthma and allergic diseases^29^. IRFs function in a network, interacting in a synergistic or antagonistic manner in conjunction with other transcription factors and adaptor molecules, and the subsequent signalling pathway determines functional outcome^41^. Recent years have brought substantial advances in our understanding of GRN and their control of cell differentiation and immune function^29, 41, 54^. However, there is currently a lack of evidence to support the application of these advances in the prediction of the outcome of immune stimulation determined by specific tissue disease states.

Dissecting the complexity of a GRN experimentally is challenging. However, computational modelling offers a promising way to approach the problem. It can provide a mathematical framework for modelling the resting state of signalling systems, including disease-specific steady states, predicting the cell and system behavior during prolonged exposure to signalling stimulus, and the outcome of multiple signalling events^20, 59^. Computational analysis of high throughput data, resulting in the network inference and dynamical simulation of the behaviour of a transcription factor network, has been shown to provide meaningful insights into the mechanisms of signal integration within a dendritic cell^19, 22^.

As predictive modelling of regulatory networks can greatly improve data analysis and data – driven hypothesis generation, a broad spectrum of mathematical formalism has been developed, allowing network modelling at different levels of detail. Quantitative continuous methods such as ordinary differential equations (ODEs), model the rate of change of each component in the network and provide detailed quantitative information regarding the networks dynamics^60^. ODEs can be used for modelling small scale GRN^22, 23, 43, 61^. For example, Hoffmann’s group demonstrates use of ODE in mass action kinetic model of chosen elements of the NFκB molecular network to achieve comprehensive characterization of the relationship between the resting state and the cellular response to stimulation^20, 22, 62^. They identified distinct temporal profiles of the activity of the central node kinase IKK or transcription factor NFκB^61^ and modelled the temporal control of the specificity of a response^63^. However, ODEs require comprehensive knowledge of kinetic parameters, which are unknown for most networks, and therefore their applicability is limited^64–66^. Furthermore, ODE modelling is computationally expensive, and therefore not suitable for large size networks^67^.

In contrast, qualitative logic-based models, such as Boolean networks^68^ and Petri nets^25, 45, 69^, do not depend on quantitative data but rather on the structure of the network along with a set of logical constraints. Qualitative regulatory networks can be built from local experimental observations or knowledge-based information (gene-gene or gene-protein interactions)^45, 46, 70^. The main advantage of qualitative networks is finite numbers of possible states making predictions about the dynamics of biological regulatory systems possible despite the lack of kinetic information. Despite being far less reliant on knowledge of rate constants than ODEs, SPN improve the quantification of Boolean networks, increasing the level of detail and faithfulness to reality, yet still preserving the ability to model large networks with relatively high speed (review: ref. 60). SPNs parametrised with discrete experimental data allows insights into the trends of molecules’ activity-levels in response to an external stimulus^24^. In our work, application of the SPN formalism, utilizing Boolean logic, allowed us to reconstruct the molecular interactions within a key gene regulatory network.

As with most models, the model proposed here is reductionist in its nature. The link between a gene’s expression and protein function is subject to complex post-transcriptional/translational regulation, which potentially limits the inference of transcriptomics data with respect to functional cellular/tissue outcomes. As a qualitative network our model does not include complex relationships between transcription factors and DNA, merely indicating that the interaction takes place. This reflects the combinatorial nature of this process, where interaction with any expressed transcriptional partner is theoretically possible, and thus stochastically modeled^42, 71^. Similarly, the model assumes that the expression levels of the output genes directly translate to the protein concentrations, which underpin the interactions with T lymphocytes and cell effector responses. However reductionist, the latter assumption has been recently justified by the work of Csardi and colleagues, whose noise-robust analyses reveal that mRNA levels explain more than 85% of the variation in steady-state protein levels^72^.

A reductionist approach was necessary to initiate the modelling and be able to derive a workable diagram of the GRN, when cells are stimulated with two opposing, ‘clean’ biological signals, rather than being exposed to a complex signalling from whole tissue. This allowed us to correctly predict a previously unreported outcome of immune stimulation based on the limited input information from a gene expression experiment in primary human LC.

Moreover, our approach not only corroborated the hypothesis that gene regulatory networks are universal and can be inferred from analysis of different cell populations, but also allowed correct prediction of an outcome of T lymphocyte stimulation by LCs based on the expression values of relatively few network components. As further validation of the approach, *in silico* modelling yielded correct prediction of perturbation of LCs function when the cells were exposed to a PI3Kγ inhibitor in the context of the intact epidermis. This prediction relied on the assumption that when applied to the whole tissue the PI3Kγ inhibitor interacts directly with LCs. As demonstrated extensively by others, PI3Kγ, in contrast to isoforms α and β, is preferentially expressed in cells of the immune system^73–75^ and the systemic effect of PI3Kγ inhibition in animal models is observed solely in immune cells^76^. Importantly, evaluation of dendritic cell function in the PI3Kγ^−/−^ mice demonstrated, that the knock-out mice had a selective defect in the number of skin LCs^77^ and showed a defective capacity to mount contact hypersensitivity and delayed-type hypersensitivity reactions^77^. Therefore, even though the epidermis harbours other leukocytes, including tissue-resident T lymphocytes^78^, which can similarly be affected by the inhibitor, our experiment corroborated the assumption, and demonstrated, that the effect of PI3Kγ inhibition in human epidermis can be mediated by LCs.

The low resolution of logic-based models imposes limitations on their predictive power. Nevertheless the presented work is in line with findings by others^24, 46^ and confirms that the correct prediction of a network’s dynamic behavior can be obtained without need for extensive experimentation and computationally expensive parameter estimation.

The comparative analysis of transcriptomics data from human LCs exposed to the contrasting epidermal signals, TNFα and TSLP, allowed us to determine the transcriptional programmes induced by the two cytokines. It has been becoming increasingly clear that *in vitro* culture can regulate the transcriptome as well as the function of cultured cells^79–81^. The direct comparison of effects of TNFα and TSLP on cells cultured in otherwise identical conditions allowed us to identify genes differentially regulated by these two cytokines, while the differences induced by the culture conditions were removed by the maSigPro algorithm. The most significant differences were discovered in the genes encoding the ability of LCs to process and present antigens. Maturation of LCs in the presence of TSLP resulted in impaired capacity to activate antigen-specific cytotoxic T cells, compared with TNFα-matured LCs. This suggests a role for TSLP in mediating the impaired CD8 T cell responses which may be of particular relevance for atopic diseases such as asthma and atopic dermatitis which are characterized by pre-disposition to viral infections^6, 82–84^. Indeed, we and others have shown that LCs are extremely potent inducers of efficient CD8 T cell activation and anti-viral immunity^2, 3, 13, 85^. Even though recent reports suggest a role of antigen exchange between LCs and subsets of dermal dendritic cells, the importance of LC has been demonstrated in murine and human systems, including their role in mediating anti-HSV immune responses through antigen uptake and processing^86, 87^. Furthermore, *in vivo*, HSV infections principally target keratinocytes (through HSV nectin-1 expression)^88^, and induce keratinocyte apoptosis in the epidermis. Therefore, in early infections, LCs uptake and processing of HSV antigens from apoptotic keratinocytes is likely to be critical.

The proposed model provides a proof of concept, to demonstrate that computational modelling of a specific immune network can predict functional outcomes of immune responses based on experimentally derived transcription levels of selected key molecular hubs. Applying this reductionist approach allowed us to determine the effect of altered cytokine signalling, as would be found in human epidermis under different conditions, and predict the impact on immune responses using easily available data (i.e. gene expression levels).

In this case a high epidermal concentration of TSLP in the milieu of atopic dermatitis would be expected to impair skin immunity against viral infection through IRF signalling pathways, which may be relevant to eczema herpeticum, and may also provide a further rationale for anti-TSLP therapy, or even targeting of IRF, in susceptible individuals.

The validation of the model has so far been limited to the *in vitro* approach, allowing investigations of the LC:T cell interaction in a controlled system. While allowing ease of manipulation, this system does not reflect complex signaling events and cell interactions *in vivo*. In order to be able to use it for the design and testing of therapeutic perturbations, it would be necessary to characterise the disease-related steady state of LCs, and validate the outcome of stimulation predicted by the model at both local (skin) and systemic level, iteratively developing the model to correctly represent the observed outcomes.

We envisage that the outlined approach can provide a platform for many future studies of human immunity, utilising data from individual transcriptomic analyses to provide predictions of how molecular interventions may alter cellular phenotype based on the actual gene expression patterns in an individual. Such comprehensive analyses ultimately enable inferring the influence of the disease state on the cellular response to stimulation. This in turn can determine the outcome of immune responses in health and in disease, and offers the possibility of predictive *in silico* testing of the effectiveness of therapeutic interventions.

## Methods

### LC isolation and culture

Skin specimens and blood samples were acquired from healthy individuals after obtaining informed written consent with approval by the Southampton and South West Hampshire Research Ethics Committee in adherence to Helsinki Guidelines. Primary cutaneous DCs were isolated as described previously (5). Briefly, following dispase (2 U/ml, Gibco, UK) digestion of epidermal sheets, migratory LCs were harvested after 48 h culture of epidermal fragments. Low density cells were enriched using density gradient centrifugation (Optiprep 1:4.2, Axis Shield, Norway) and purified with CD1a+ magnetic beads according to manufacturer’s protocol (Miltenyi Biotec, UK). Epidermal and dermal DCs were purified with magnetic beads according to manufacturer’s protocol (epidermal cells: CD1a+, dermal cells: CD11c+, Milenyi Biotec, UK). Cells were assayed for yield and cell viability, and unstimulated cells (time 0, 250,000/cell type/donor) were harvested immediately. For analysis of changes in gene expression upon activation, LCs were stimulated with TNF-α or TSLP (25 ng/ml, 15 ng/ml respectively, Miltenyi Biotec, UK) for 2, 8 and 24 h (250,000 cells/cell type/donor/time point). For analysis of epidermal explant culture, epidermal sheets from 6 mm biopsies were cultured with epidermal cytokines as described above, and the RNA was isolated for qRT-PCR gene expression gene analysis from the LCs were harvested 48 h later. For pulsing with a nominal CD8+ T cell epitope, LCs were incubated with 10 μM of a proGLC peptide, containing 9 aminoacid HLA-A2 restricted EBV-derived epitope (FNNFTVSFWLRVPKVSASHLEGLCTLVAML; Peptide Protein Research, UK) for 18 h, with TNFα added at 6 h, and washed thoroughly before co-culture with T cells. EBV-peptide-specific T cell line was expanded as described in detail previously^2, 3^. For PI3Kγ inhibition human epidermal biopsies (6 mm) were exposed to the effects of AS605240 at the non-toxic dose 0.1 uM or 1 μl DMSO (control diluent). Migratory LC were co-cultured with allogeneic naïve CD4 T cells. Secretion of IFNγ and IL-4 was measured in an ELISpot experiment as per manufacturer protocol, (Mabtech, Sweeden), after 6 days of priming and re-stimulation with phytohemagglutinin (PHA) (n = 6, in triplicate).

### Microarray data analysis

RNA was isolated using RNeasy mini kits (Qiagen, UK) as per the manufacturer’s protocol. RNA concentration and integrity was determined with an Agilent Bioanalyser. All the samples had a RIN > 7.0 and were taken forward for labelling. Gene expression analysis was carried out using the Human Genome U-219 Affymetrix platform (LC stimulation with cytokines) or Human Gene ST 1.1 Affymetrix platform, Affymetrix ATLAS system, for cell migrating post PI3Kγ inhibition. Expression data were normalised using the Robust Multichip Average (RMA) package within the Affymetrix expression console package and annotated. After an initial QC check, the data was taken forward for analysis. To identify genes regulated by exposure of LCs to TNFα and TSLP, a cutoff threshold 0.05 of Bayesian estimation of temporal regulation^36^ for genes showing ×1 log(2)-fold difference between the gene expression level at a given time point and time 0 control. Probesets differentially regulated by TNFα and TSLP, were identified using MaSigPro algorithm (24) p < 0.05. Using network analysis tool BioLayout *Express* ^3D^ ^38^, a transcript-to-transcript correlation matrix was calculated for 527 probesets fulfilling the criteria above, where each column of data was derived from a different sample (donor/cell type/condition) and each row of data represents an individual probeset (25). A non-directional network graph of the data was generated for a Pearson correlation coefficient of *r* ≥ 0.80. In this context, nodes represent individual probesets (genes/transcripts) and the edges between them Pearson correlation coefficients between individual probesets above the threshold value. The network graph was then clustered into groups of genes sharing similar profiles using the MCL algorithm within the BioLayout *Express* ^3D^ tool with an MCL inflation value set to 1.7, as reported previously (26). Gene set enrichment analysis was performed using the “functional annotation clustering” tool, (similarity threshold 0.5, multiple linkage threshold 0.5, EASE:1.0 and Benjamini correction) from DAVID (27) and ToppGene^40^ web-based analysis tools and confirmed by detailed direct analysis using Gene Expression Atlas (http://www.ebi.ac.uk/gxa/). All microarray data used for these studies are available in GEO, Accession No.: TNFα and TSLP: GSE49475. PI3Kγ: GSE94247.

### Model Assembly

To identify components for the IRF GRN a systematic search in PubMed was performed, as summarised in Table S1. Separate searches have been performed for each combination of terms. From the returned papers, 82 unique original papers were identified, describing regulation of gene expression by IRF and their transcription partners (Table S2). The experimental findings within each listed reference papers have been analysed to identify the stimulus, cell type, biological process controlled by IRF (including lists of genes identified by ChIP-seq analysis), the interaction partner, and the DNA binding sequence. This information has been categorised as the network components: input node, transmission node, output node and mode of interaction.

To facilitate the network assembly, the data has been structured into an interaction database, containing entries for: (1) stimulus, (2) interaction partner A, (3) interaction partner B, (4) mode of interaction, (5) DNA sequence, (6) gene transcription/biological process. This information is shown in Table S2. The interaction database was sorted and analysed to identify experimental findings validated by multiple reports. To be included into the network architecture, the interaction had to be confirmed by two independent reports. If any referenced publication reported only part of the information (e.g. only interaction between IRF and transcription partner, but not the DNA sequence) the lacking information have been inferred from the complementing reports.

To convert the database of interactions into a Boolean network, a checkerboard of interactions between the network elements have been assembled (Table S3), assigning for each interaction gate “and”, where both components are essential, “or”, when one of the components is essential or “inhibition”.

The network diagram was constructed using yED (yFiles, Germany) following the mEPN notation^46^, allowing computational modelling of concurrent systems. For a detailed description of a diagram assembly please refer to Livigni *et al*.^45^. Signalling Petri Nets are an extended application of stochastic Petri nets (SPN) originally described by Ruths *et al*.^24^. This method integrates elements of a Boolean network simulator with the synchronized Petri net model for the network represented using the classic Petri net view of places and transitions. In brief, the signalling Petri Net algorithm models the stochastic ‘flow’ of variable numbers of tokens through the network, solely determined by the initial input values and the network architecture. The tokens are assigned to the GRN entry transitions, and represent quantities of the biological molecules, in case of the IRF-GRN, the levels of expression of the transcript. The amount of tokens assigned at the entry (the network initial marking set-up) can be either theoretical, representing a binary on/off expression levels, corresponding to a biological knock-out situation, or derived from biological experiment, and representing the quantity of the transcript measured in cells.

### IRF GRN model parametrization and *in silico* simulations

The network diagram has been drawn in a mEPN notation^24, 46^, allowing computational modelling of concurrent systems. When formerly constructed as a bipartite graph, nodes represent biological entities and transitions represent biological interactions. The abundance of a molecule at any given network node can be represented by the placement of tokens. Edges connecting the nodes and transitions determine the direction of the token flow through the diagram, representing the progress of the biological process. The detailed description of network assembly can be found in the Supplementary Material and methods. To validate the graph reachability and correct prediction of the postulated biological effect in the presence of one or many TF, initial marking (number of tokens in the network entry nodes) has been set up as a theoretical value either 0 or 100 for every possible combination of the entry nodes: *IRF1*, *IRF4*, *IRF8*, AP1-binding and ETS-binding. To test the network behavior in physiological conditions, the initial marking of the SPN has been set as per the levels of expression from microarray data analysis, Table S6. Simulations were executed using BioLayout *Express* ^*3*D^, 100 time blocks, 500 runs.

## Electronic supplementary material

Supplementary material
