# Supplementary material for: Petri Net computational modelling of Langerhans cell Interferon Regulatory Factor Network predicts their role in T cell activation
Source: Sci Rep. 2017 Apr 6;7:668. doi: 10.1038/s41598-017-00651-5 (PMC5428800; doi:10.1038/s41598-017-00651-5)
Supplement: Supplementary file 1 — Supplementary material [file 41598_2017_651_MOESM1_ESM.pdf]

Petri Net computational modelling of Langerhans cell Interferon Regulatory Factor Network predicts their role in T cell activation.

Authors: Marta E. Polak, MSc, PhD*[1,2], Chuin Ying Ung[1], MD, Joanna Masapust[1], MSc, Tom C. Freeman, PhD[3,4] , Michael R. Ardern-Jones, BSc, FRCP, DPhil[1,4]

[1] Clinical and Experimental Sciences, Sir Henry Wellcome Laboratories, Faculty of Medicine, University of Southampton, SO16 6YD, Southampton, UK

[2] Institute for Life Sciences, University of Southampton, SO17 1BJ, UK.

[3] The Roslin Institute and Royal (Dick) School of Veterinary Studies, University of Edinburgh, Easter Bush, Edinburgh, Midlothian EH25 9RG, UK

[4] These authors contributed equally to this work

***Corresponding Author:**

Dr. Marta E. Polak,

Address: Clinical and Experimental Sciences, Faculty of Medicine, University of Southampton, Southampton General Hospital, LE59, MP813, SO16 6YD, Southampton, UK

Tel: 02381205727, e-mail: m.e.polak@soton.ac.uk

SUPPLEMENTARY MATERIALS AND METHODS

Assembly of the IRF GRN diagram

Components of the GRN are represented by rectangles connected by arrows indicating molecular interactions (blue arrow: synergism, red arrow: inhibition). GRN output (i.e. immunological function) is presented in octagons on the right side of the diagram. The diagram is drawn in a Petri Net notation, where the interacting elements of GRN (nodes, gene transcripts) are interspaced with transitions (vertical black lines, and black diamonds). The diagram captures the combinatory nature of immune activation, depending on the levels of expression, timing and interactions between the regulatory elements. The flow of the signal through the diagram can be modelled mathematically using experimental or theoretical data and visualised in BioLayout Express^{3D}. The abundance of a molecule at any given network node can be represented by the placement of tokens. Edges connecting the nodes and transitions determine the direction of the token flow through the diagram, representing the progress of the biological process.

The network assembly has been done in the following steps:

Introducing network components (rectangles, nodes) and interactions (black diamonds, transitions):

a. Input nodes: IRF 1,4, and 8, and transcription partners grouped as ETS or AP-1 family. Assumption: IRF can bind with any TP from the ETS family. There are 28 members of ETS family, and 5 AP-1 binding transcription factors. Only the transcription partners exceeding 150 RMA normalised expression level in the human skin DC microarray dataset were included in the diagram.

b. DNA binding sequences: EICE, ISRE, AICE

c. Genes controlled by each IRF or IRF-TP heterodimer (ChIP-seq data, only the genes exceeding 150 RMA normalised expression level in the human skin DC microarray dataset and related to activation of T cell activation by DCs were included in the diagram).

d. Output nodes (octagons): Regulated genes

e. Output nodes (octagons): Biological processes

f. Interactions: black diamonds

Connecting the network components with edges to represent the identified interactions.

a. IRF, IRF TP, and corresponding DNA binding sequence were connected with stimulatory edges (black arrows) including all possibilities detailed in the Table S3 (e.g. IRF8 can bind to EICE with ETS, to AICE with AP-1 or ISRE with IRF1), preserving the and/or logic (i.e. IRF8 cannot bind to ISRA without IRF1: transition “and”, IRF 1 can connect to ISRA either on its own or hetero-dimerised with IRF8: transition “or”). The Boolean logic gates “and” and “or” have been recreated using two nodes to transition (“and”) and two transition to node (“or”) (Signal flow through “and” ad “or” gate is presented in Figure S2)

f. Genes identified by ChIP-seq analysis of IRF1,4, and 8 were associated with the DNA binding sequence, and with the output biological process. Assumption: controlling IRF homo/heterodimer determines DNA binding sequence. If a gene can be controlled by two transcription factors/two DNA sequence (e.g. IL18 via IRF1 or IRF8/ETS complex) both possibilities were included in the diagram.

Adding entry transitions for input nodes: (transitions: black bars)

a. An entry transition was added before each entry node to allow setting up initial marking of the network and input of the numerical data.

Converting diagram edges into appropriate interactions (stimulatory: black arrows, inhibitory: red open diamonds)

a. Each edge drawn is initially a black stimulatory edge. To convert the interaction to an inhibitory, the arrow was replaces with an open diamond shape end. For clearer visualization the inhibitory edges are colored red.

SUPPLEMENTARY FIGURE LEGENDS

Figure S1. Regulation of transcription factor expression in human LCs by epidermal cytokines.

A list of probesets encoding transcription factors filtered to contain one probeset per gene, and above 150 expression value have been curated from the whole transcriptome dataset, using and the transcription factors activated during LC activation with epidermal cytokines were identified using (LIMMA,(*28*)). Expression values (a,b, d,f) and fold change differences (c,e,g) are visualised for top transcription factors expressed at base line (a) up-regulated (b-e) and differentially regulated (f,g) by epidermal cytokines are shown. T0 DDC used for comparison in (a) have been isolated as previously published {Polak, 2014 #2259}.

Figure S2. Network of IRF and their transcription partners regulates transcriptional programmes of dendritic cells.

a) Model of Interferon Regulatory Factors gene regulatory network (GRN) in LCs, assembled based on a systematic literature review, depicting; IRF, transcription partners, DNA sequences and transcribed genes arranged left to right. Each interaction has been confirmed by two independent reports in myeloid cells. Components of the GRN are represented by rectangles connected by arrows representing molecular interactions (black arrow: synergism, red arrow: inhibition). GRN output (i.e. immunological function) is presented in octagons on the right side of the diagram. The diagram is drawn in a Petri

net notation, where the interacting elements of GRN (nodes, gene transcripts) are interspaced with transitions (vertical black lines, and black diamonds). The diagram captures the combinatory nature of immune activation, depending on the levels of expression, timing and interactions between the regulatory elements. The flow of the signal through the diagram can be modelled mathematically using experimental or simulated data and activity flow visualised in BioLayout Express3D.

b-e) Effect of signal transmission through "and" and "or" Boolean gates

Petri Net network motifs demonstrating the principles of signal flow through "and" (b,d) and "or" (c,e) gates with input from single (b,c) and multiple (d,e) transitions. Initial network marking = 100, token accumulation after gate are shown in the right column, 100 time blocks, 500 runs, simulation under the conditions of standard distribution.

Figure S3: Genes expression profiles in transcriptional programmes "A" and "B" match the *in silico* prediction

a) *In silico* profiles of gene expression in programmes "A" and "B" , measured at the output node when the input nodes are marked as per the gene expression values during LCs stimulation with TNF-α and TSLP, Signalling Petri Nets: BioLayout *Express*3D, 100 time blocks, 500 runs. **b**) Expression profiles of individual genes in "Programme A" as measured in the microarray experiment. **c**) Expression profiles of individual genes in "Programme B" as measured in the microarray experiment.

Figure S4: Ability of LC to cross-present antigens is modified by TNFα and TSLP.

Activation of antigen-specific CD8+ T cells by medium (white), TNFα (grey), TSLP (black) and a combination of TNFα and TSLP (black checkerboard grey) matured LCs, pulsed with a long peptide antigen requiring cross-presentation, IFN-γ production measured in co-culture ELISpot assay, n=2 in triplicate, mean +/- SE.

Figure S5: Effect of PI3K-γ inhibitor on the ability of LC migrating from epidermal biopsies to polarise naïve CD4 T cell responses.

Human epidermal biopsies (a) were exposed to PI3Kγ inhibitor or control media for 48h. The ability of migratory LCs (b) to polarise adaptive immune responses was predicted *in silico* (c,d, BioLayout Express3D, 100 time blocks, 500 runs) and measured in vitro (e,f) IFNγ (e) and IL-4 (f) measured by Elispot, n=6, in triplicate, mean ± SEM shown. Control media (black bars) or PI3Kγ inhibitor [1µMol] (grey bars). Experimental p values: Paired t-test for means

SUPPLEMENTARY TABLES

Table S1: Search strategy to identify components of the IRF GRN network

Table S2: Interaction database

Table S3: Boolean gates

Table S4: Genes regulated by IRF1,4 and 8: ChIP-seq analysis

Table S5: Genes regulated by expression programme "A" and "B" in the IRF-GRN

Table S6: Experimentally measured expression values at 0h (0-8 time block),2h (9-32 time block),8h (33-75 time block),and 24h (76-100 time block) converted to parametrisation values for each GRN entry nodes.

Table S7: Experimentally measured expression values for input nodes in LC migrating in the presence or absence of PI3K-gamma inhibitor, AS605240, average of n=2.

Supplementary Figure 1

a Top 40 TF expressed in LCs at T0

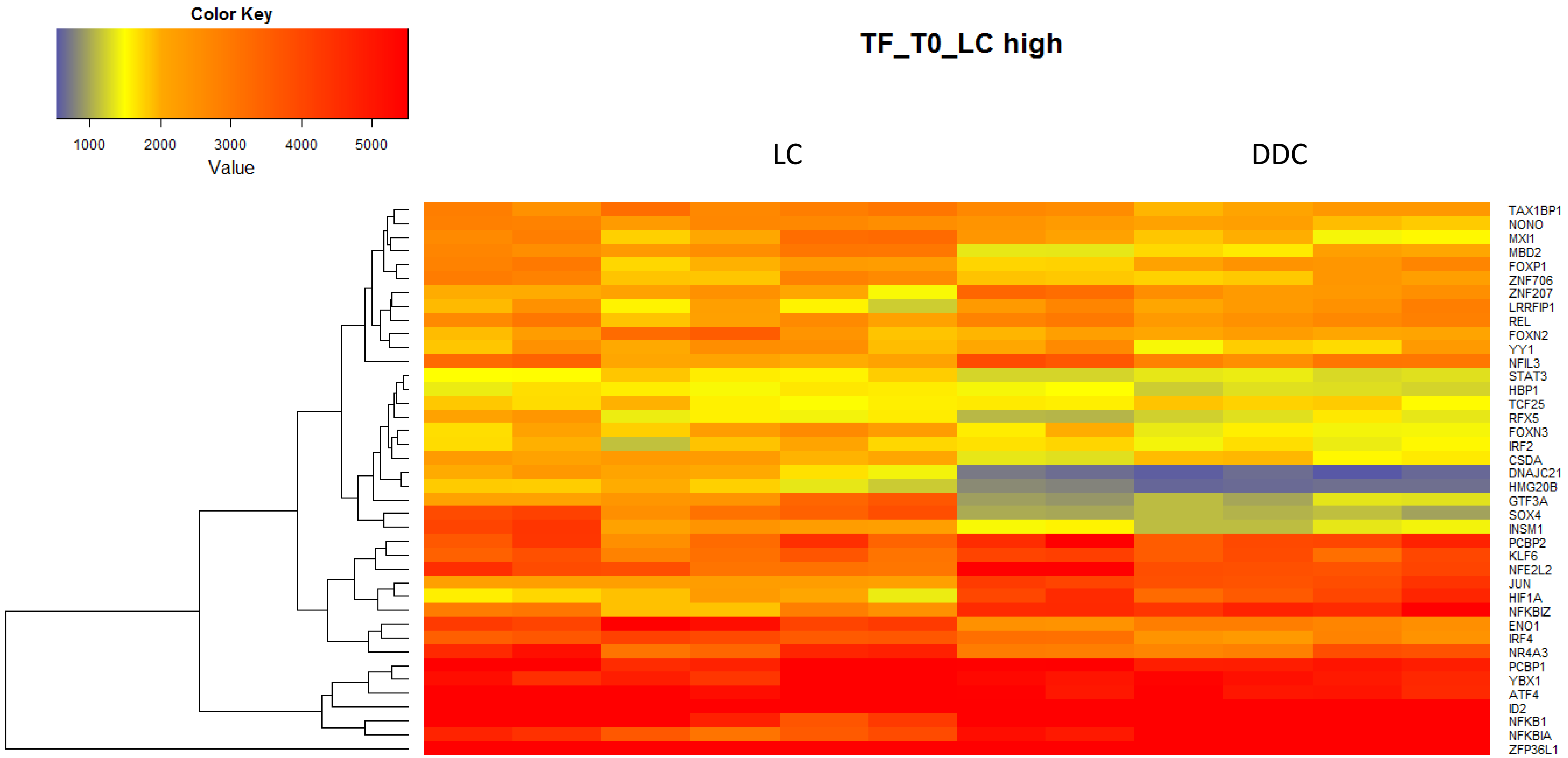

Supplementary Figure 1

b Top 40 TF up-regulated in LCs at 2h stimulation with TNFa

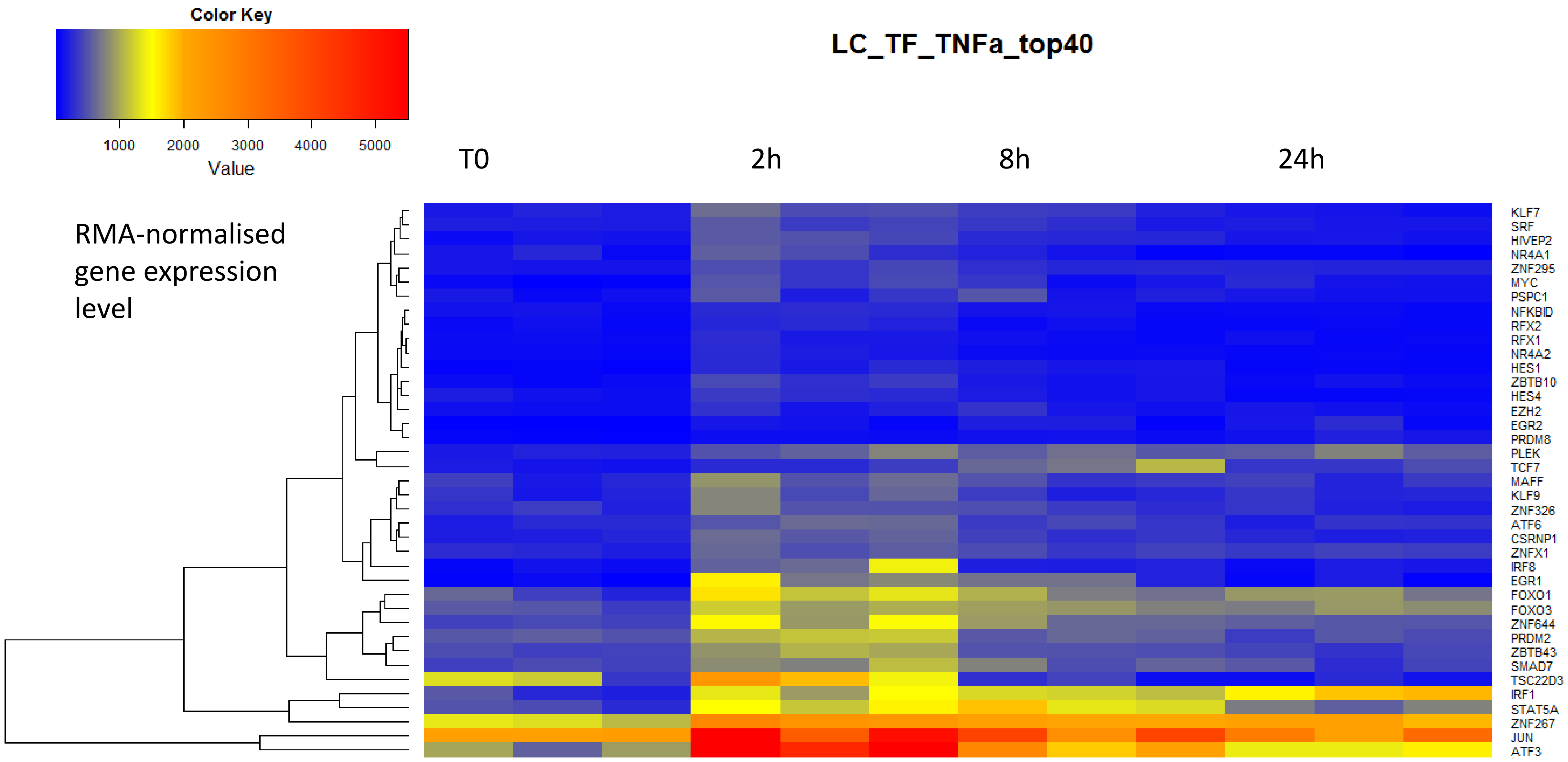

Supplementary Figure 1

c Top 40 TF up-regulated in LCs at 2h stimulation with TNFa

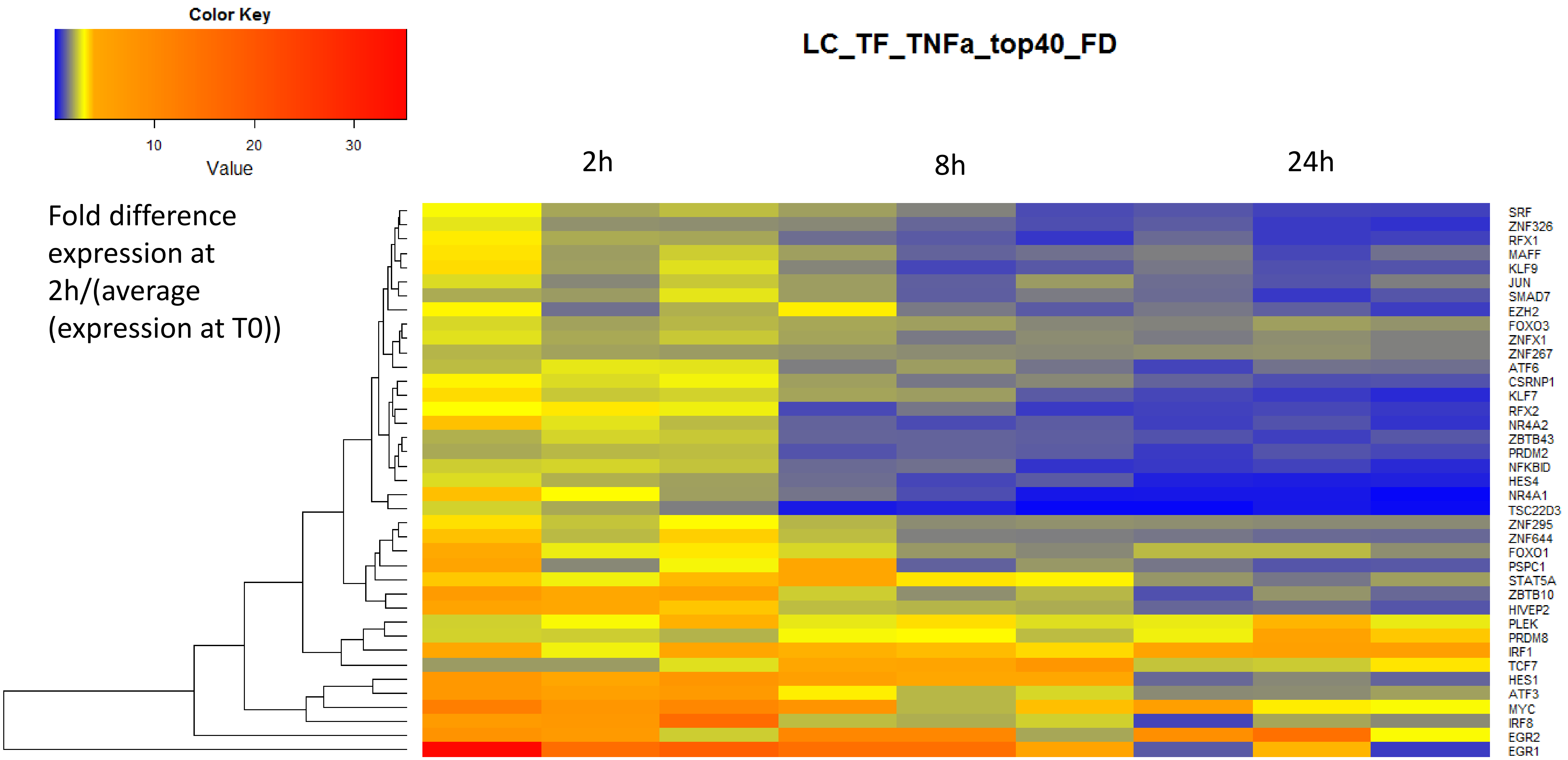

Supplementary Figure 1

d Top 20 TF induced by TNFa & Top 20 TF induced by TSLP

RMA-normalised gene expression level

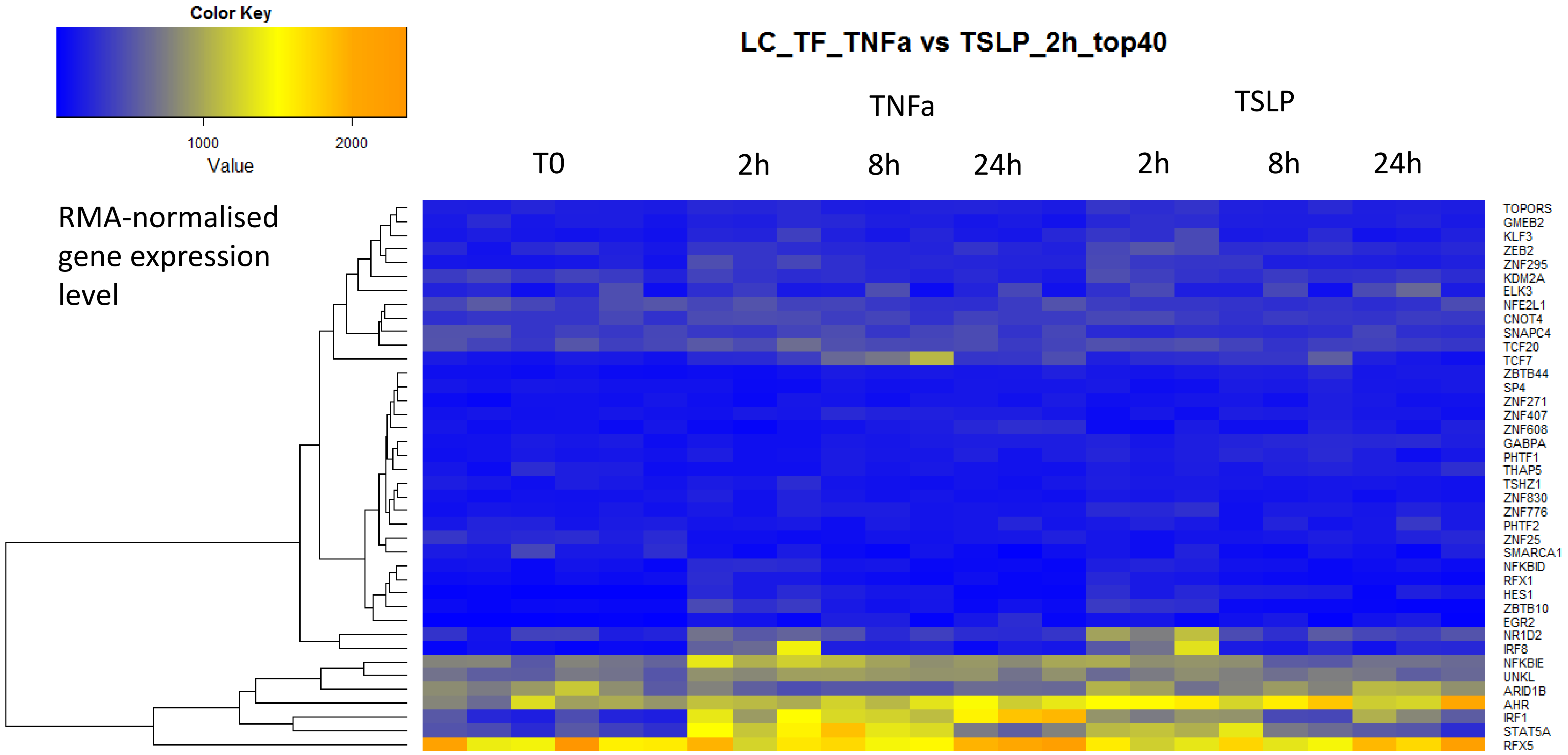

Supplementary Figure 1

e Top 20 TF induced by TNFa & Top 20 TF induced by TSLP

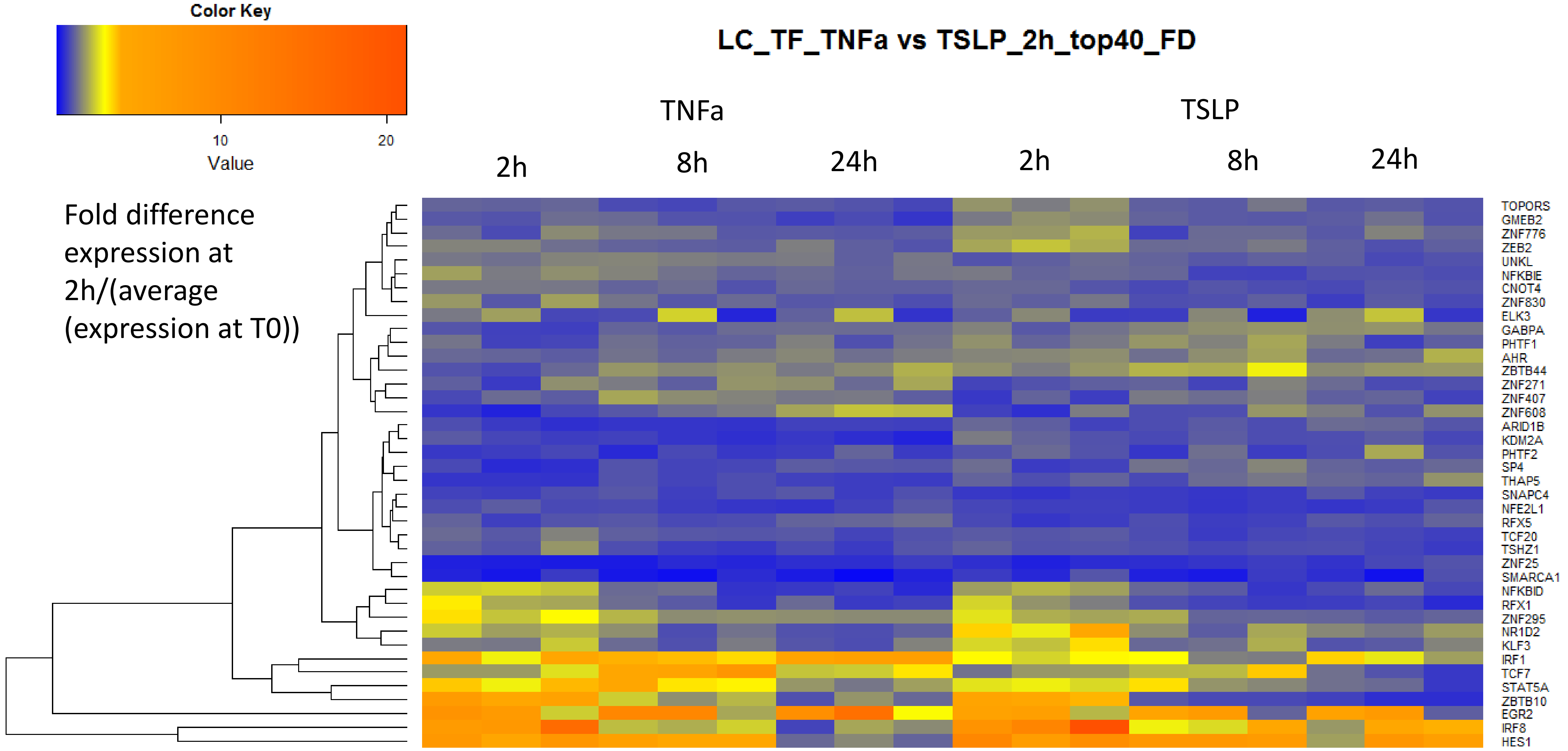

Supplementary Figure 1

f Top 20 TF induced by TNFa & Top 20 TF induced by TSLP

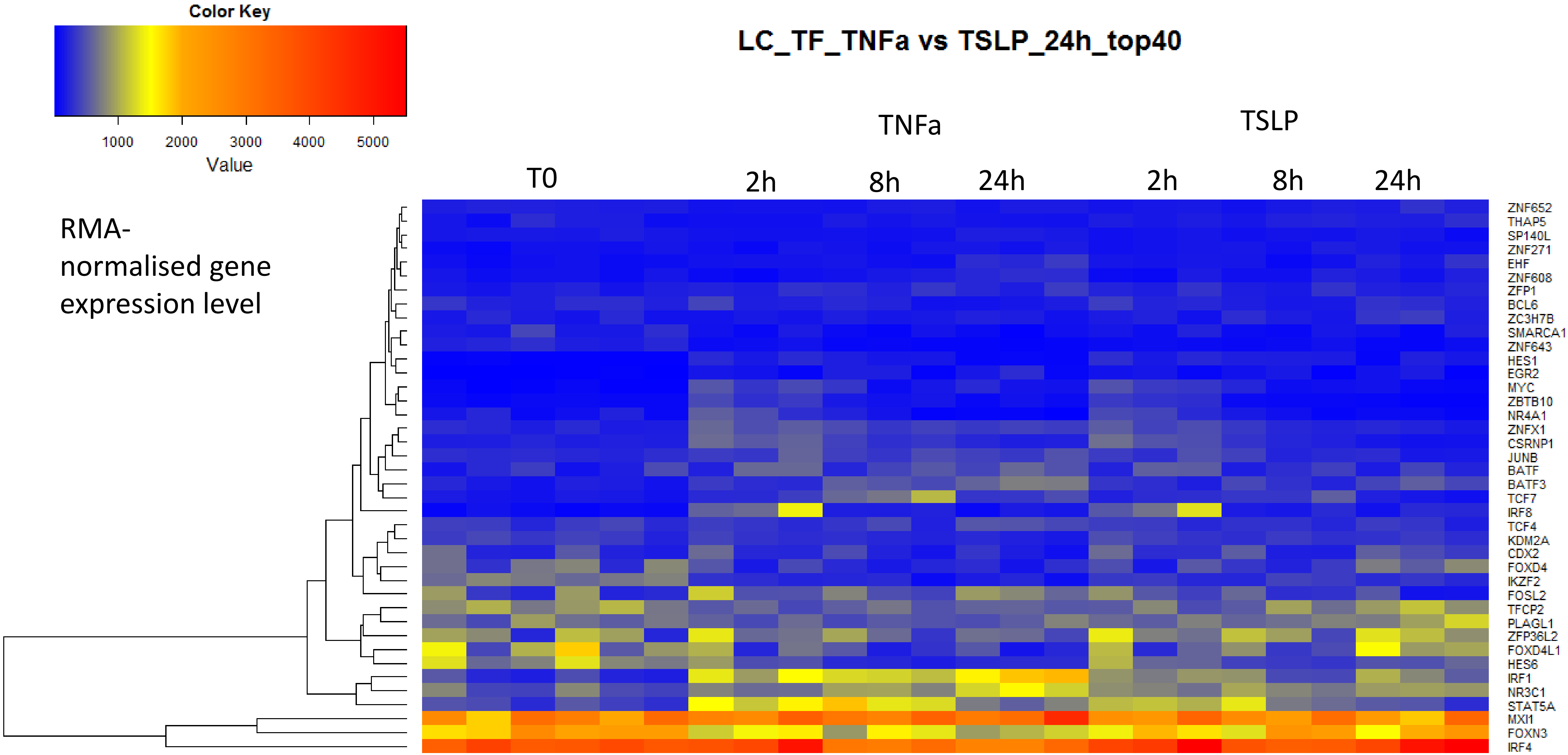

Supplementary Figure 1

g Top 20 TF induced by TNFa & Top 20 TF induced by TSLP

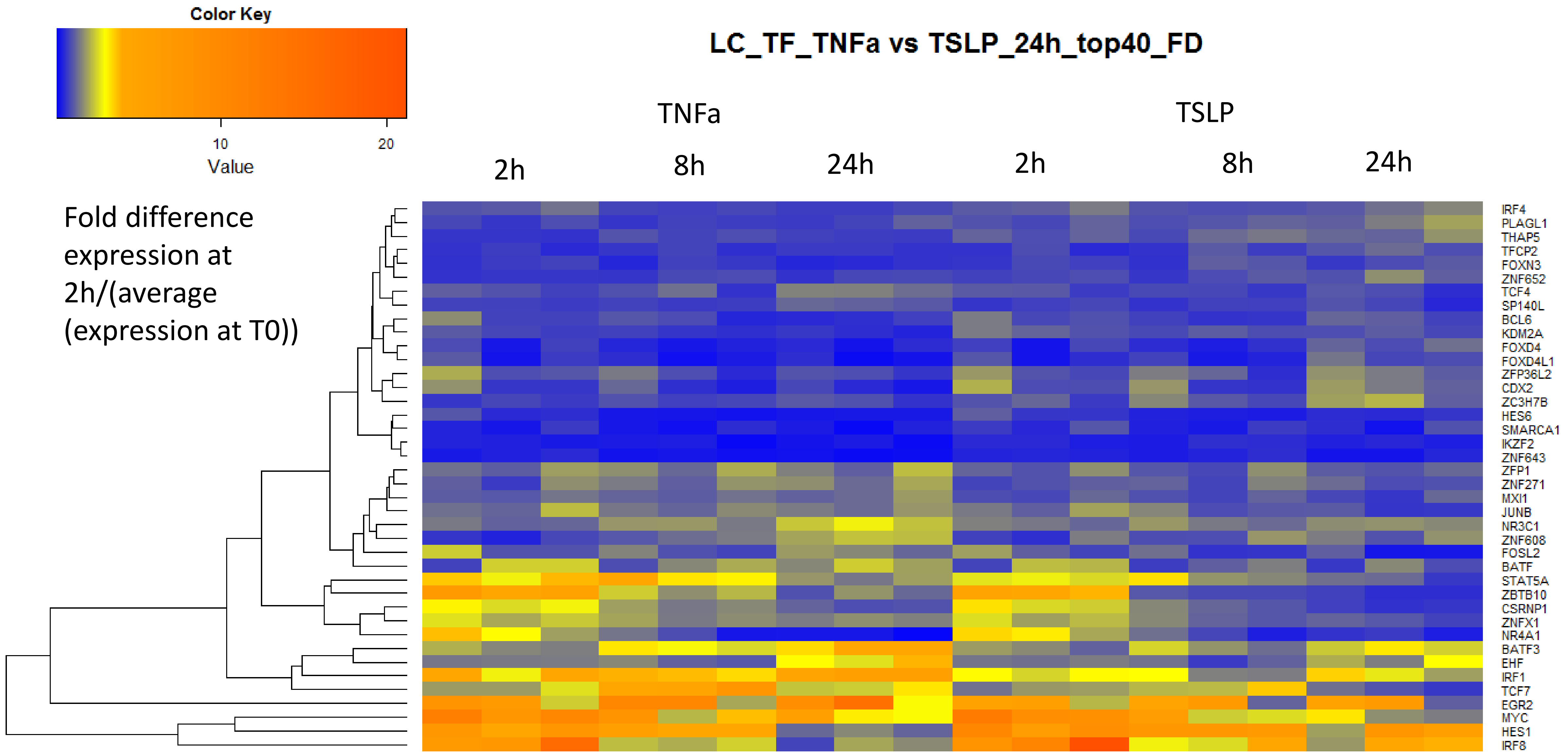

Supplementary Figure 2. Network of IRF and their transcription partners regulates transcriptional programmes of dendritic cells.

a

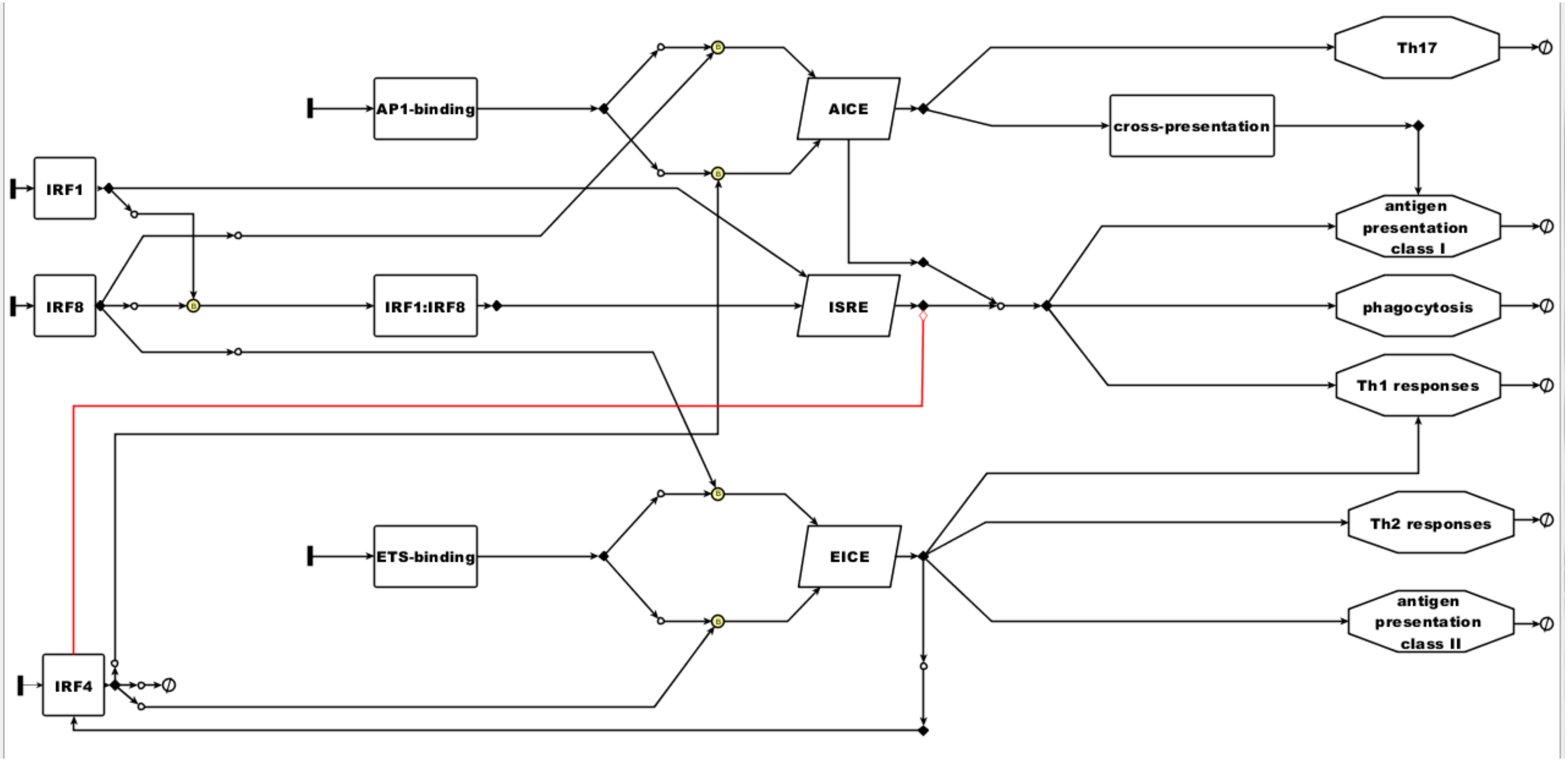

Supplementary Figure 2: Boolean networks: and/or gates

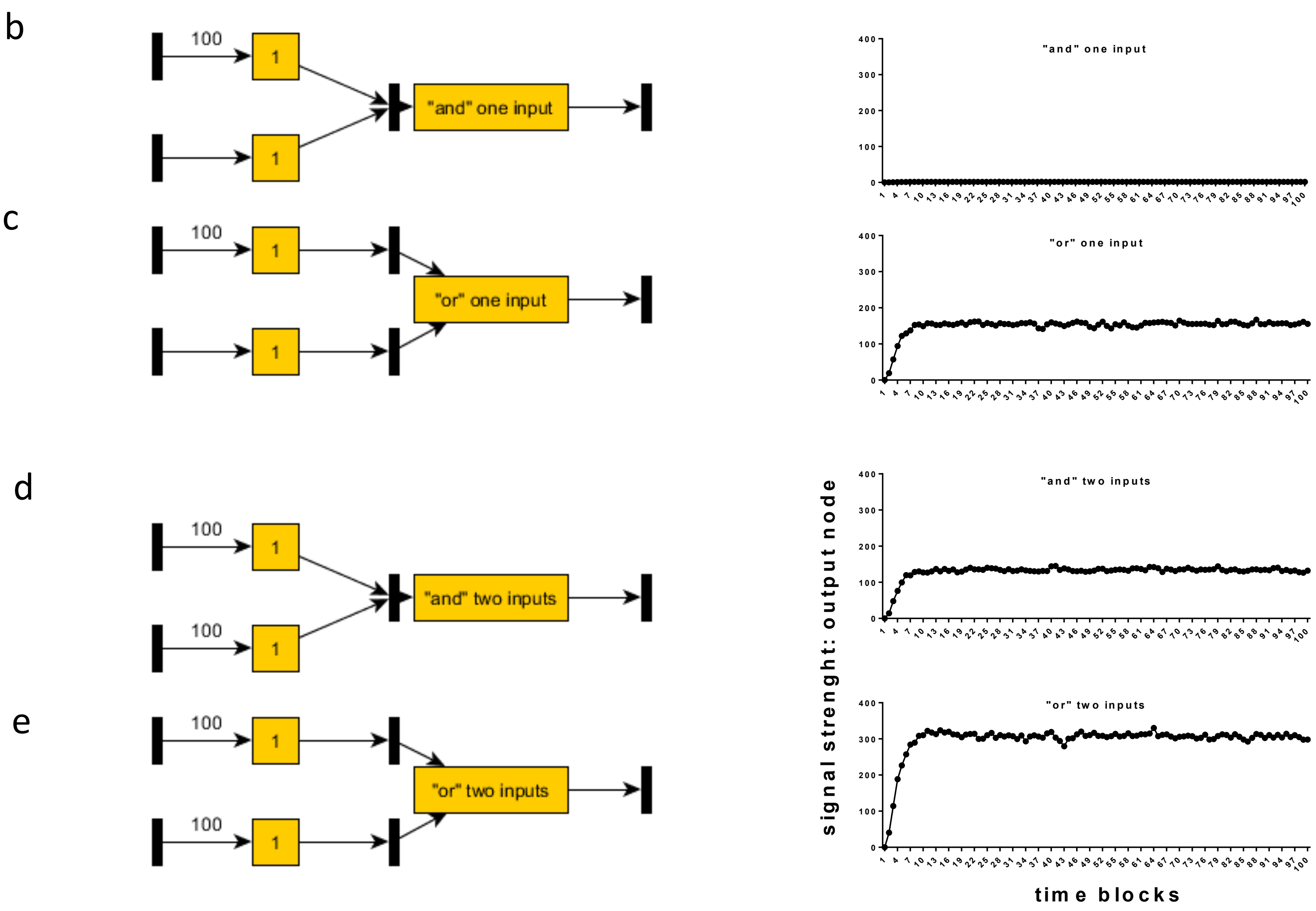

Supplementary Figure 3:
In silico profiles of genes involved in programme "A" and "B"

a

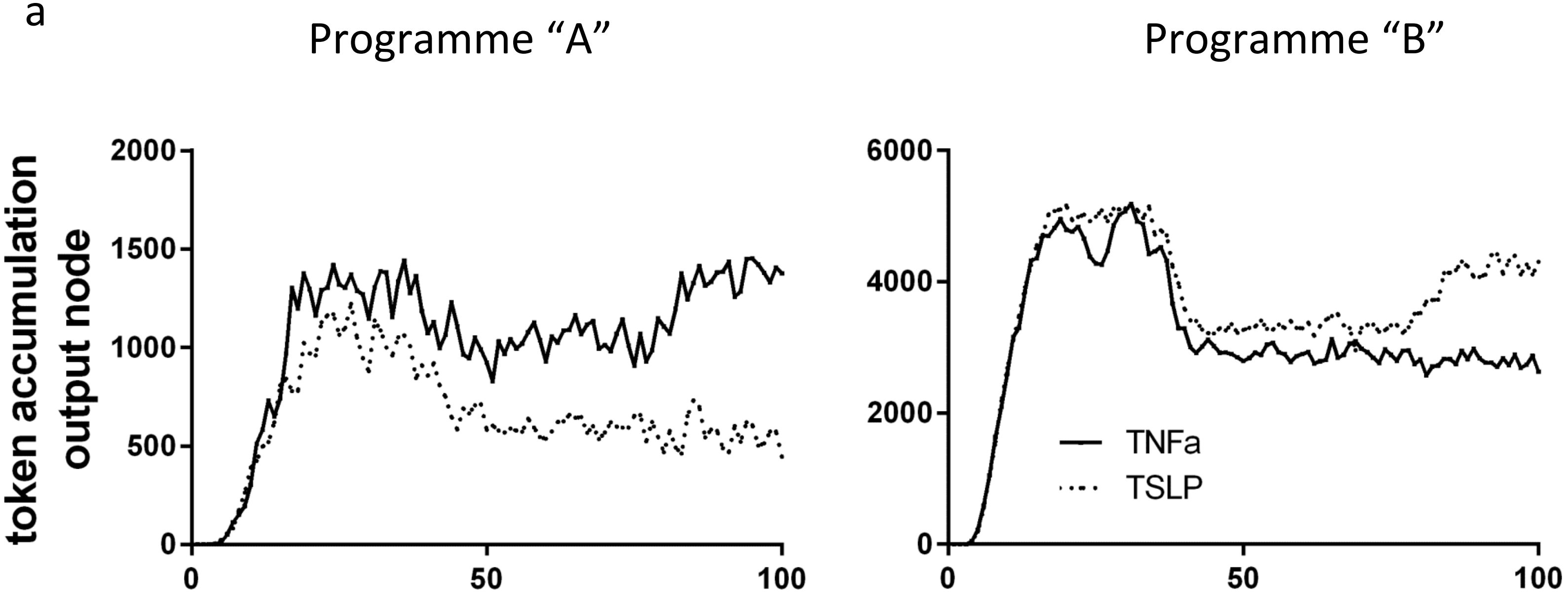

b

Programme “A” : genes up-regulated by TNF-α

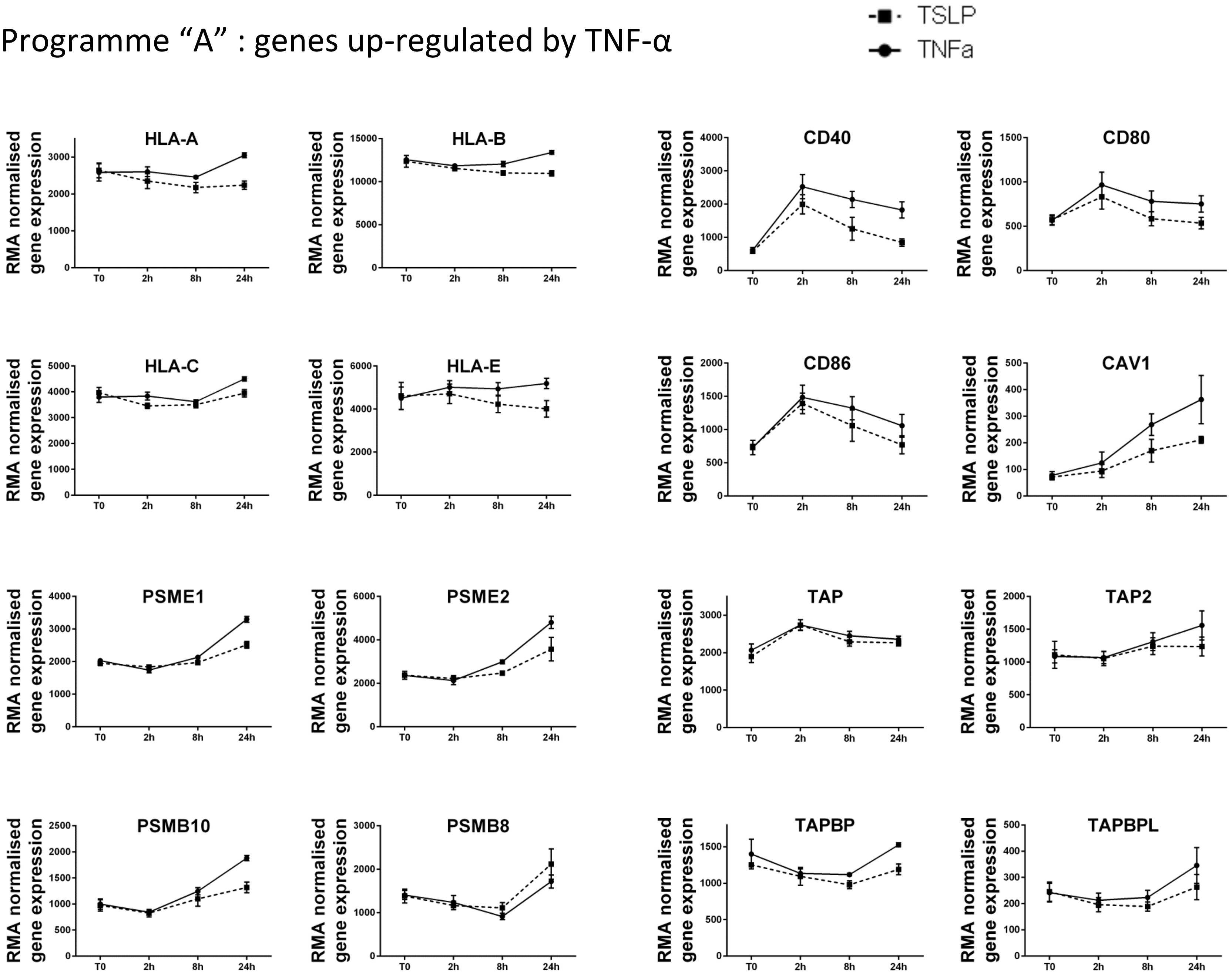

c

Programme “B” : genes regulated in simillar manner by TNF-α and TSLP

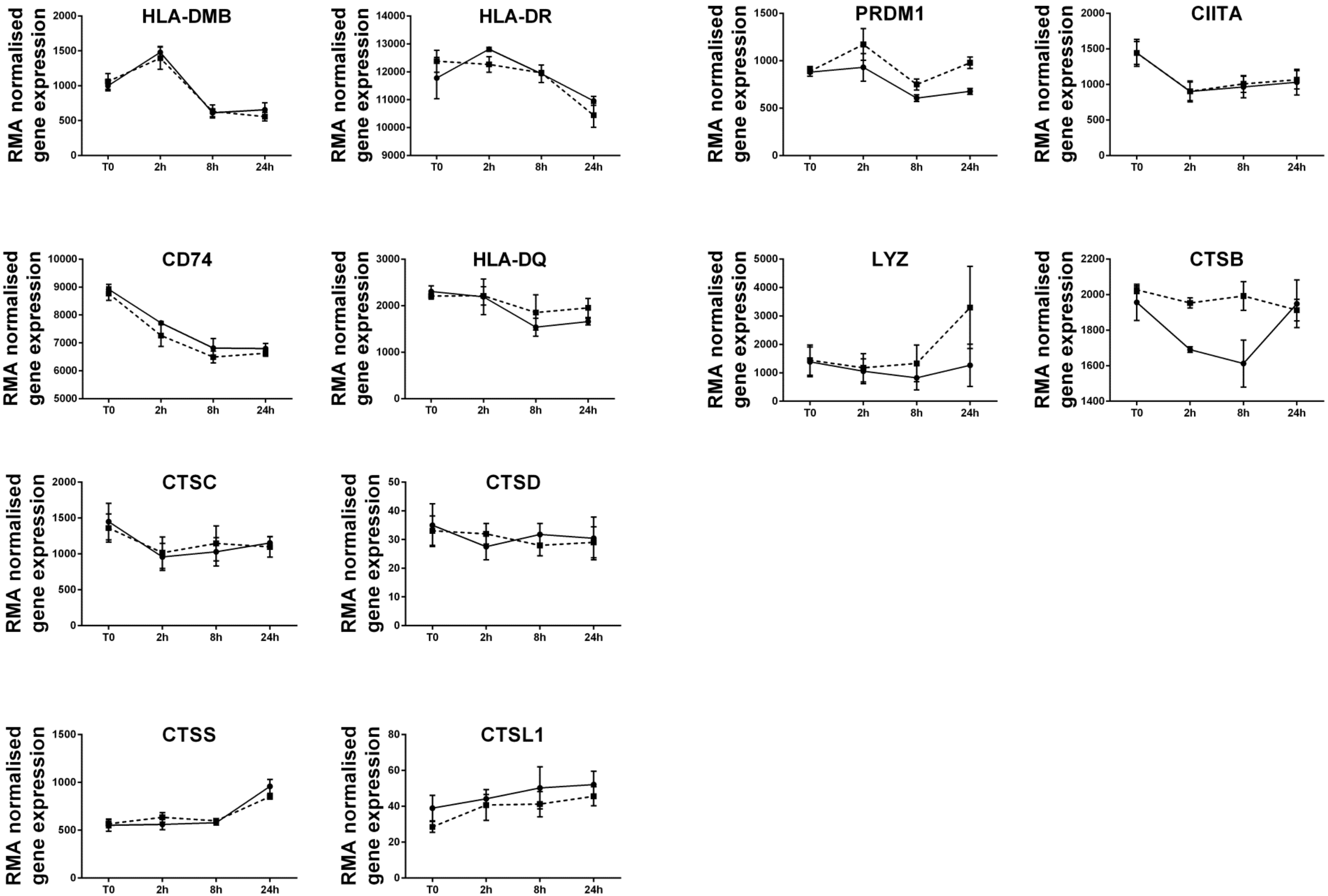

cytokines

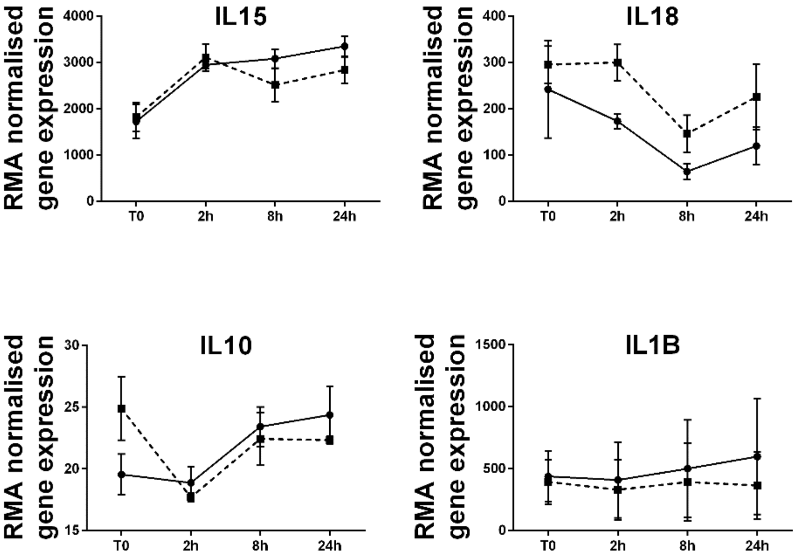

ERAP

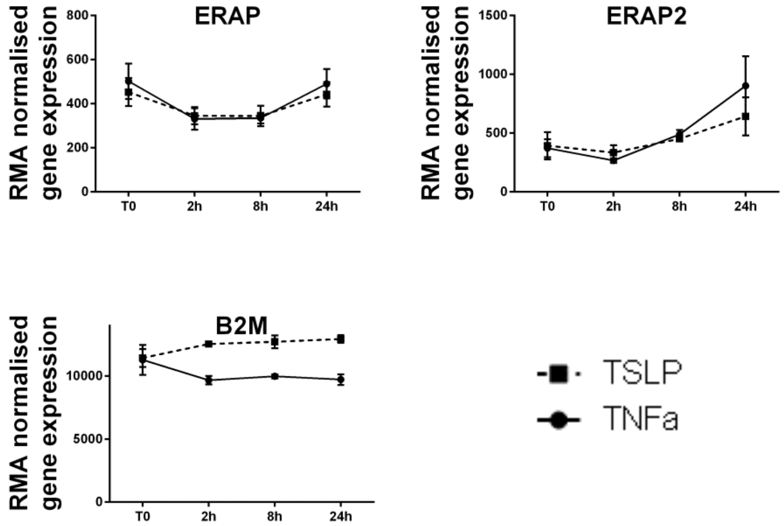

Supplementary Figure 4. Ability of LC to cross-present antigens is modified by TNFα and TSLP.

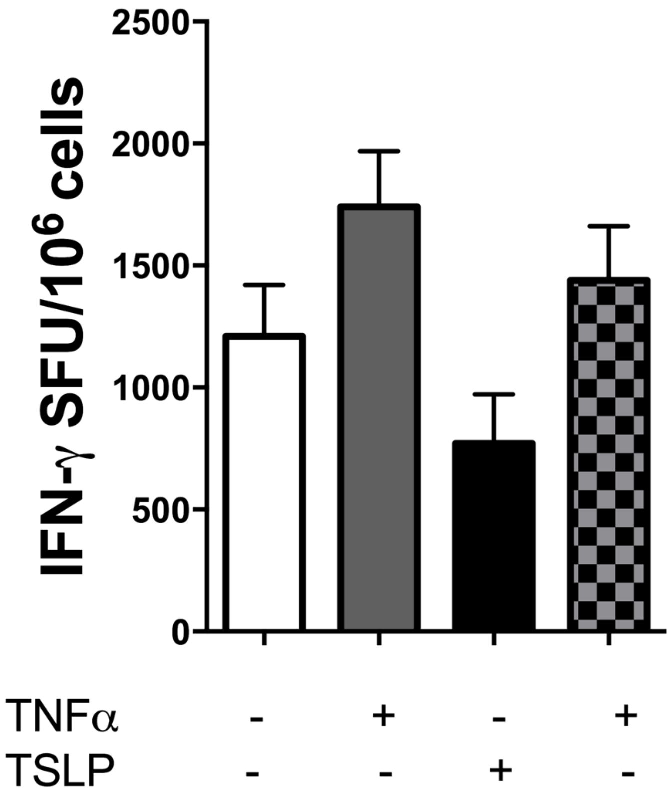

Supplementary Figure 5. Effect of PI3Kγ inhibitor on the function of LC migrating from epidermal biopsies.

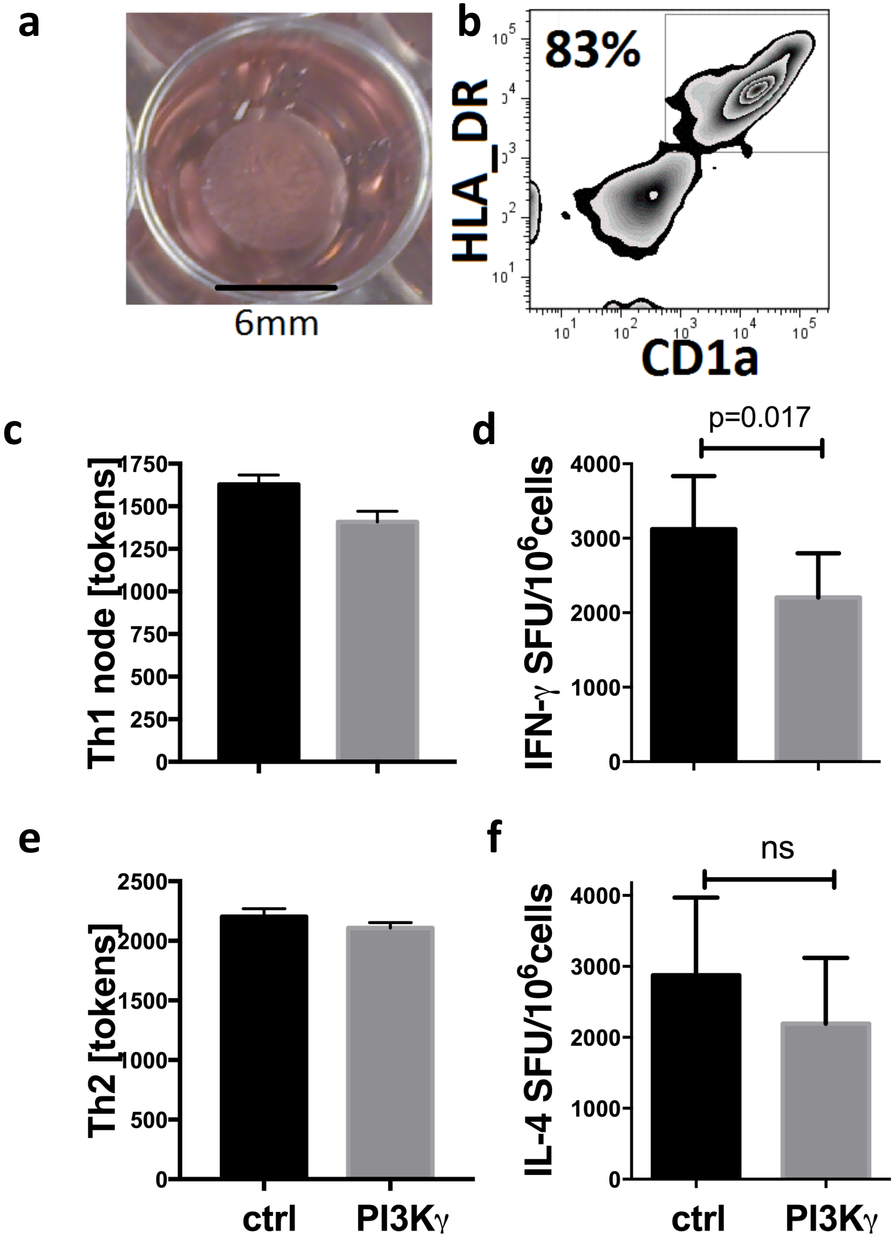

Table S1. Search strategy to identify components of the IRF GRN network

search term	number of publications
"Interferon regulatory factor" or IRF and antigen presentation	71
"Interferon regulatory factor" or IRF and dendritic cell and T cell stimulation	22
"Interferon regulatory factor" or IRF1 or IRF4 or IRF8 and *transcripton partner* as per the transcription partner list	510
Interferon regulatory factor or IRF1 or IRF4 or IRF8 and ChIP-seq	15

Table S2. Interaction database

Citation	pubmed id	cell type	Stimulus	partner A	interaction	Partner B	DNA sequence	outcome
Hildner Science 2008	19008445	DC (mouse)		BATF3	essential			cross-presentation
Hildner Science 2008	19008445	DC (mouse)		BATF3	essential			anti-viral responses
				BATF3		IRF4/8???	AICE?	cross-presentation and CD8 responses
Ma JBC 1997	9099678	nucleic acid level	IFNg priming for LPS	ETS2		?	ETS2 - site, complex F1	IL12p40
				ETS2		?		IL12p40+>Th1
Roy JI 2015	25957166	macrophages	IFNg	IRF1	synergy	BATF2	IRF1 binding	Th1
Marecki JI 2001	11359842	fibroblasts (transf)		IRF1	synergy	IRF4/PU.1	ISRE/EICE	IL1B
Marecki JI 2001	11359842	fibroblasts (transf)		IRF1	synergy	IRF8/PU.1	ISRE/EICE	IL1B
Shi Gene 2001	21803131	monocytes		IRF1				antigen processing to class I
Gabrielle J Leukocyt Biol 2006	16966383	DC (mouse)		IRF1		inhibits		immunological tolerance
Gabrielle J Leukocyt Biol 2006	16966383	DC (mouse)		IRF1	essential	essential		immunological activation - CD8 Th1
Casola J Virol 2001	11413310	RSV infected alveoral epithelial cells		IRF1			ISRE	CCL5
Elser Immunity 2002	12479817	T cells		IRF1	inhibitory		IRFB, A, C sites (IL-4 pro	suppression of IL4
Karmann J Exp Med 1996	8691131	HUVEC	CD40, TNFa, IL1b	IRF1		??ATF-2/cJun/CREB		VCAM1, ICAM1,MHC class I
Kirchhoff FEBS 1999	10215868			IRF1		NFkB	PRD1-3, not 4	IFN-b
Fujita Nature 1989	2911367			IRF1			PRD1, PRD3	IFN-b
Fujita PNAS 1989	2557635		IFNg, TNFa, IL1, PolyI:C	IRF1				IFN-b
Kumatori JBC 2002	11781315			IRF1		STAT1	-100 GAS & -88 ISRE	gp91 phox
Eklund JI 1999	8805641			IRF1				gp91 phox
Saura J Mol Biol 1999	10356322	macrophage line (mouse)	IFNg and TNFa	IRF1	physical change of prom	NFkb		iNOS
Dror Mol Immunol 2007	16597464	macrophages		IRF1	binding	IRF8		Th1
Dror Mol Immunol 2007	16597464	macrophages		IRF1	binding	IRF8		iNOS
Gabrielle J Leukocyt Biol 2006	16966383	DC (mouse)		IRF1 null	essential			immunological tolerance
Masumi Mol Cell Biol	10022868	Mo/MF lines		IRF1, IRF2	essential	PCAF	ISRE	endogeneous IRF (only 1 and 2) recruit histone acetylases to enhance transcription
Masumi Mol Cell Biol	10022868	Mo/MF lines		IRF1, IRF2	essential	CBP/p300	ISRE	
Masumi Mol Cell Biol	10022868	Mo/MF lines		IRF1, IRF2	essential	GCN5	ISRE	
interaction				IRF1	essential and sufficient for ISRE and Th1		ISRE	Th1
interaction				IRF1	essential and sufficient for ISRE and CD8		ISRE	CD8 Tcells/ag presentation in class I
Marecki JI 2001	11359842	macrophages	LPS	IRF4		PU.1		IL1B
Marecki JI 2001	11359842	macrophages	IFNg+ LPS	IRF4		PU.1		IL1B
O'Reily JBC 2003	12676954	macrophages		IRF4		PU.1		repression of CD68
Ahyi JI 2009	19592658	T cell (mouse)		IRF4		PU.1 ?		Th2 - low cytokines
Glasmacher Science 2012	22983707			IRF4		BATF in the absence of I	AICE	Th17
Matsuyama Nucl Acid Res 1995	7541907		antigen-receptor interac	IRF4			ISRE	MHC class I
Brass 1996	8824592	B cells		IRF4		PU.1	EICE	induces B cell differentiation
Brass 1996	8824592	B cells		IRF4				represses IFN-inducible proliferation
Eisenbeis 1995	7797077	B cells		IRF4		PU.1	ISRE/EICE	mutual co-activation
Escalante 2002	12372320			IRF4		PU.1	AAxxGGAA IECS/EICE?	mutual co-activation
Kwon Immunity 2009	20064451	T cells	IL21	IRF4		STAT3	TTTC	PRDM1 (BLIMP1, T cell differentiation)
Honma PNAS 2008	18836070	T cell (naive)	TCR	IRF4	competition	IRF1	competitive	programming of Th responses
Yamagata 1996	8657101	T cells		IRF4	suppression	IRF1	GAGGAAACGAAACC	binding, suppression of IRF1
Li Nature 2012	22992523	T cells	IL21, CD3-CD28	IRF4		BATF/JUN	AICE	IL10 in Th17 responses

Yoshida 2005	16172134	HeLa	inserted	IRF4 (dominant negative	suppression	IRF1	tandem GAAA (ISRE?)	repression of transcription TRAIL
Yamamoto Plos One 2011	22003407	macrophages		IRF4		IECS		cytokines, IL6, IL12b
Vander Lugt Nat Immunol 2014	24362890	DC (mouse)		IRF4		EICE		antigen presentation class II
Lehtonen JI 2005	16272311	MoDC, MoMF		IRF4		STAT4		DC lineage
Lehtonen JI 2005	16272311	MoDC, MoMF		IRF4		p50, p65		DC lineage
Lehtonen JI 2005	16272311	MoDC, MoMF		IRF4		PU.1		IRF4
Williams Nature Com 2014	24356538	BMDC (mouse)		IRF4		PU.1		IL10, IL33, Th2
Williams Nature Com 2014	24356538	BMDC (mouse)		IRF4				Th1 - no effect
Tussiwand Nature 2012	22992524	BMDC (mouse)		IRF4		BATF, 2,3	AICE	CD8a DC differentiation
Sciammas Immunity 2006	16919487	B cells		IRF4 high	induction	PMDR1		PMDR1, antagonising plasma cell differentation
Ochiai Immunity 2013	23684984	B cells		IRF4 high/sustained	dimerisation, low affinity	IRF4	ISRE	antagonising plasma cell differentation
Ochiai Immunity 2013	23684984	B cells		IRF4 high/sustained	dimerisation, low affinity	IRF4		PMDR1
Ochiai Immunity 2013	23684984	B cells		IRF4 low/transient		PU.1	EICE	
Ochiai Immunity 2013	23684984	B cells		IRF4 low/transient		BATF	AICE	
Ochiai Immunity 2013	23684984	B cells		IRF4 low/transient			EICE/AICE	plasma cell differentiation
Meraro JI 2002	12055236	immune cells		IRF4		PU.1	EIRE	ISRE-dependent genes
Ochiai Immunity 2013	23684984	B cells		IRF4 low/transient		PU.1		PMDR1
Rosenbauer Blood 1999	10590072			IRF4			ISRE	inhibitory
interaction				IRF4	cooperation	PU.1 (ETS)	EICE (=IECS??)	Th2
interaction				IRF4	cooperation	BATF (AP1)	AICE	Th17
interaction				IRF4	cooperation	PU.1 (ETS)	EICE (=IECS??)	CD4 Tcells/Class II presentation
interaction				IRF4 high	inhibition	IRF1	ISRE	inhibition of IRF1
Marecki JI 2001	11359842	fibroblasts (transf)		IRF8		PU.1	ISRE?? Surely EICE	IL1B
Tamura Blood 2005	15947094	macrophages (mouse)		IRF8				endocytosis and lysosyme
Huang JBC 2007	17200120	U937		IRF8		PU.1		NF1 (cytokine induced proliferation of myeloid cells)
Huang JBC 2007	17200120	U937		IRF8		IRF2/PU.1		NF1 (cytokine induced proliferation of myeloid cells)
Bovolenta PNAS 1994	8197182	Jurkat		IRF8		IRF1	ISRE	
Bovolenta PNAS 1994	8197182	Jurkat		IRF8		IRF2	ISRE	
Bovolenta PNAS 1994	8197182	Jurkat		IRF8			ISRE	inhibits binding of IRF9
Yamamoto Plos One 2011	22003407	macrophages		IRF8				cytokines, IL6, IL12b
Liu JBC 2004	15489234	macrophages (mouse)		IRF8		IRF1	*ISRE?*	IL18
Kim JI 1999	10438937	macrophage line (mouse)	LPS	IRF8		PU.1	*EICE*	IL18
Eklund JBC 1998	9593745			IRF8	PU.1 essential for the co	IRF1		CYBB = gp91 phox
Eklund JI 1999	10570299	cell lines - reporter assays	IFNg	IRF8	PU.1 essential for the co	IRF1		CYBB = gp91 phox, NCF2 = gp67 phox
Tamura Immunity 2000	10981959	Tot2 progenitor, mice		IRF8			ISRE	macrophage lineage
Tamura Immunity 2000	10981959	Tot2 progenitor, mice		IRF8		PU.1	EICE	macrophage lineage
Masumi FEBS 2002	12417340	macrophages (murine line)	IFNg priming for LPS	IRF8		IRF1	ISRE-like	IL12p40
Masumi FEBS 2002	12417340	macrophages (murine line)	IFNg priming for LPS	IRF8		IRF1	ETS/NFkB no effect	IL12p40

Tussiwand Nature 2012	22992524	BMDC (mouse)		IRF8		BATF, 2,3	AICE	CD8 T cell responses
Meraro JI 2002	12055236	immune cells		IRF8		PU.1	EIRE	ISRE-dependent genes
Smith MA JBC 2011	21216962	MoDCs, DC (mouse), THP1	LPS	IRF8/IRF4	PU.1	EICE		CIITA leading to PRDM1
Rosenbauer Blood 1999	10590072	macrophages		IRF8			ISRE	inhibitory
Weish 1994	7526889			IRF8	competition	IRF1	ISRE	MHC class I
Brass 1996	8824592	B cells		IRF4				represses IFN-inducible proliferation
Nelson 1993	7678054	N-Tera2.		IRF8	inhibits		ISRE	Interferon-induced genes
Salem 2014	25122610	dendritic cell		IRF8				dendritic cell function, CD4 and CD8 T cell activation
interaction				IRF8	cooperation	PU.1 (ETS)	EICE	CD4/Th1
interaction				IRF8	cooperation	BATF (AP-1))	AICE	CD8 T cell responses
interaction				IRF8	cooperation	IRF1	ISRE	Th1
Smith MA JBC 2011	21216962	MoDCs, DC (mouse), THP2	LPS	p65, SP1		SP1 and NFKB binding		CIITA leading to PRDM1
Weiss JI 2012	22896628	BMDC (mouse)	Lactobacillus	phagosomal processing, PI3K and MyD88		IRF1, IRF3/7		IFNb
Sciammas Immunity 2006	16919487	B cells		PMDR1	induction	IRF4 low		AICDA, plasma cell differentiation
Smith MA JBC 2011	21216962	MoDCs, DC (mouse), THP3	LPS	PRDM1	competition	IRF8	EICE	CIITA silencing
Crotty Nat Immunol 2010	20084069	B cells, T cells		PRDM1		BCL6		antagonistic interactions
Gyori Nat Immunol 2010	14985713	B Cells		PRDM1				histone lysine 9 dimethyltransferase G9a
Kuo and Calame JI 2004	15494505	B cells		PRDM1	competition	IRF1	GAAAG	IFNb
Yu 1999	10713181	B cells		PRDM1		Histone deacetylase		repression of transcription
Su 2008	19124609	B cells		PRDM1		Histone deacetylase		repression of transcription
Piskurich Nat Immunol 2000	11101876	B cells		PRDM1		CIITA		antagonistic interactions
interaction				PRDM1	competition	IRF4/8	EICE	repression of CIITA
interaction				PRDM1 induced by high	competition	IRF1	ISRE????	negative feedback loop?
Chang Immunity 2005	15963784	T cell (mouse)		PU.1				Th2 - low cytokines
Ahyi JI 2009	19592658	T cell (mouse)		PU.1		GATA3		Th2 - high cytokines
Walsh Immunity 2002	12433372	hematopoietic progenitors		PU.1		Gata-2		negative regulation of macrophage/mast cell differentiation
Smith MA JBC 2011	21216962	MoDCs, DC (mouse), THP3	LPS	PU.1		PU.1 binding		CIITA leading to PRDM1
Suzuki PNAS 1998	9600921	T cells, Mo, B cells		PU.1				gp91 phox
Ma JBC 1997	9099678	nucleic acid level	IFNg priming for LPS	PU.1			ETS2 - site, complex F3	IL12p40
Heidari Gene 2012	22659071	Neuronal cell lines					IRF/ETS binding site	Caveolin
Du PNAS 1994	7972056			NFkB		ATF-2 or cJUN/ATF	PRD2, PRD4	IFN-b
Du Cell 1993	8374955			NFkB		ATF-2 or cJUN/ATF	PRD2, PRD4	IFN-b
Cheng Science Signalling 2011	21343618	BMDM macrophages	IFNb and LPS	p50	repression	IRF3/IRF9	G-IRE, guanine rich IRE	early response, Tap1, IL15
Casola J Virol 2001	11413310	RSV infected alveoral epithelial cells		C/EBP	NF-kB	IRF,CREB/AP-1		CCL5 - multiple cis-regulation required
Heinz Mol Cell 2010	20513432	macrophages		C/EBP	CCAAT enhancer binding	PU.1	CCAAT	lineage determination
Heinz Mol Cell 2010	20513432	macrophages		C/EBP and AP1		PU.1		lineage determination
Eklund JI 1999	10570299	cell lines - reporter assays	IFNg	CBP (CREB binding protein)		IRF8/PU.1/IRF1		CYBB = gp91 phox, NCF2 = gp67 phox
Skalnik JBC 1991	1885602			CUX1	CCAAT displacement protein (repressive)		CCAAT	gp91 phox
Luo Skalnik JBC 1996	8798551			CUX1 absent		IRF2		gp91 phox
Ma JBC 1997	9099678	nucleic acid level	IFNg priming for LPS	ETS2		IRF1	ETS2 - site, complex F1	IL12p40
Ma JBC 1997	9099678	nucleic acid level	IFNg priming for LPS	ETS2			ETS2 - site, complex F2	IL12p40
Ma JBC 1997	9099678	nucleic acid level	IFNg priming for LPS	ETS2	?PU.1 - induced by IFNg	IRF1	ETS2 - site	IL12p40
interaction				TF		diverse TP		transcription partners from the same family can replace each other in the interaction

Table S3. Boolean gates

Interaction partner 1	GATE	Interaction partner 2	interaction	binding site	outcome	GATE
IRF1	and	IRF1	induction	ISRE	TH1/CD8	
IRF1	inhibition	IRF4	inhibition	ISRE	TH1/CD8	
IRF1	and	IRF8	induction	ISRE	TH1/CD8	
IRF1	not reported	AP1				
IRF1	not reported	ETS				
IRF4	and	IRF4	inhibition	ISRE	TH1/CD8	
IRF8	and	IRF8	inhibition	ISRE	TH1/CD8	OR
IRF4	and	AP1	induction	AICE	TH17	
IRF4	and	ETS	induction	EICE	TH2	OR
IRF8	and	ETS	induction	EICE	CD4	
IRF8	and	AP1	induction	AICE	CD8	OR
PRDM1	inhibition	IRF4		EICE	CD4	
PRDM1	inhibition	IRF8		EICE	CD4	OR

Table S4. Genes regulated by IRF1,4 and 8: ChIP-seq analysis

Citation	pubmed id	cell type	Stimulus	gene/ineraction	gene regulated	process
Dror Mol Immunol 2007	16597464	macrophages		IRF8	IL12p40	Activation of Th1 immune responses
Dror Mol Immunol 2007	16597464	macrophages		IRF1	IL12p40	
Dror Mol Immunol 2007	16597464	macrophages		IRF1 and IRF8	iNOS	
Dror Mol Immunol 2007	16597464	macrophages		IRF1 and IRF8	p67	
Dror Mol Immunol 2007	16597464	macrophages		IRF1 and IRF8	gp91	
Dror Mol Immunol 2007	16597464	macrophages		IRF1 and IRF8	IL-18	
Dror Mol Immunol 2007	16597464	macrophages		IRF1 and IRF8	ISG15	
Dror Mol Immunol 2007	16597464	macrophages		IRF1 and IRF8	IL-12	
Dror Mol Immunol 2007	16597464	macrophages		IRF1 and IRF8	CXCL16	
Dror Mol Immunol 2007	16597464	macrophages		IRF1 and IRF8	H28	
Dror Mol Immunol 2007	16597464	macrophages		IRF1 and IRF8	IL7R	
Dror Mol Immunol 2007	16597464	macrophages		IRF1 and IRF8	LIF	
Dror Mol Immunol 2007	16597464	macrophages		IRF1 and IRF8	MAP4K4	
Dror Mol Immunol 2007	16597464	macrophages		IRF1 and IRF8	MMP9	
Dror Mol Immunol 2007	16597464	macrophages		IRF1 and IRF8	MYC	
Dror Mol Immunol 2007	16597464	macrophages		IRF1 and IRF8	PCDH7	
Dror Mol Immunol 2007	16597464	macrophages		IRF1 and IRF8	PML	
Dror Mol Immunol 2007	16597464	macrophages		IRF1 and IRF8	SOCS7	
O'Reily JBC 2003	12676954	macrophages		PU.1	CD68	
O'Reily JBC 2003	12676954	macrophages		Fli1	CD68	
O'Reily JBC 2003	12676954	macrophages		ELF1	CD68	
O'Reily JBC 2003	12676954	macrophages		MEF	CD68	
O'Reily JBC 2003	12676954	macrophages		PU.1 and IRF4	repression of CD68	
Shi Gene 2001	21803131	monocytes		IRF1	HLA-H	Antigen presentation class I
Shi Gene 2001	21803131	monocytes		IRF1	ERAP1	
Shi Gene 2001	21803131	monocytes		IRF1	TAPBP	
Shi Gene 2001	21803131	monocytes		IRF1	PSME1	
Shi Gene 2001	21803131	monocytes		IRF1	ERAP2	
Shi Gene 2001	21803131	monocytes		IRF1	PSMB9	
Shi Gene 2001	21803131	monocytes		IRF1	TAP2	
Shi Gene 2001	21803131	monocytes		IRF1	TAPBPL	
Shi Gene 2001	21803131	monocytes		IRF1	B2M	
Shi Gene 2001	21803131	monocytes		IRF1	CD209	
Shi Gene 2001	21803131	monocytes		IRF1	PSMB8	
Shi Gene 2001	21803131	monocytes		IRF1	OAS3	
Shi Gene 2001	21803131	monocytes		IRF1	APOBEC3F	
Shi Gene 2001	21803131	monocytes		IRF1	FCGR1C	
Shi Gene 2001	21803131	monocytes		IRF1	IL29	
Shi Gene 2001	21803131	monocytes		IRF1	IL18BP	
Shi Gene 2001	21803131	monocytes		IRF1	PSEN1	
Shi Gene 2001	21803131	monocytes		IRF1	GBP1	

Shi Gene 2001	21803131	monocytes		IRF1	TLR3	
Shi Gene 2001	21803131	monocytes		IRF1	IFI35	
Shi Gene 2001	21803131	monocytes		IRF1	DHX58	
Shi Gene 2001	21803131	monocytes		IRF1	TNFRSF14	
Shi Gene 2001	21803131	monocytes		IRF1	APOL1	
Shi Gene 2001	21803131	monocytes		IRF1	ETS1	
Shi Gene 2001	21803131	monocytes		IRF1	APOBEC3G	
Shi Gene 2001	21803131	monocytes		IRF1	TNFSF13B	
Shi Gene 2001	21803131	monocytes		IRF1	IL15	
Shi Gene 2001	21803131	monocytes		IRF1	BST2	
Shi Gene 2001	21803131	monocytes		IRF1	CD274	
Tamura Blood 2005	15947094	macrophages (mouse)		IRF8	Cst3	
Tamura Blood 2005	15947094	macrophages (mouse)		IRF8	Lyzs	
Tamura Blood 2005	15947094	macrophages (mouse)		IRF8	Ctsc	
Tamura Blood 2005	15947094	macrophages (mouse)		IRF8	Psap	
Tamura Blood 2005	15947094	macrophages (mouse)		IRF8	Ctsl	
Tamura Blood 2005	15947094	macrophages (mouse)		IRF8	Pde4a	
Tamura Blood 2005	15947094	macrophages (mouse)		IRF8	Rgs2	
Tamura Blood 2005	15947094	macrophages (mouse)		IRF8	Lyn	
Tamura Blood 2005	15947094	macrophages (mouse)		IRF8	Prkcd	
Tamura Blood 2005	15947094	macrophages (mouse)		IRF8	Jak2	
Gabrielle J Leukocyt Biol 2006	16966383	DC (mouse)		IRF1	CD40	
Gabrielle J Leukocyt Biol 2006	16966383	DC (mouse)		IRF1	CD80	
Gabrielle J Leukocyt Biol 2006	16966383	DC (mouse)		IRF1	CD86	
Gabrielle J Leukocyt Biol 2006	16966383	DC (mouse)		IRF1	IL12 p40	
Gabrielle J Leukocyt Biol 2006	16966383	DC (mouse)		IRF1	IL15	
Gabrielle J Leukocyt Biol 2006	16966383	DC (mouse)		IRF1	TNFa	
Gabrielle J Leukocyt Biol 2006	16966383	DC (mouse)		IRF1	IFNg	
Ahyi JI 2009	19592658	T cell (mouse)		IRF4	IL10	
Ahyi JI 2009	19592658	T cell (mouse)		IRF4	IL4	
Glasmacher Science 2012	20064451	T cells	IL21	IRF4 and BATF	IL17	activation of Th1 immune responses
Heidari J Neuroimmunol 2011	21683457	brain			CAV1	
Honma PNAS 2008	18836070	T cell (naive)	TCR	IRF4	inhibits IL4	
Honma PNAS 2008	18836070	T cell (memory)	TCR	IRF4	promotes IL4	
Li Nature 2012	22992523	T cells	IL21, CD3-CD28	IRF4/BATF/JUN	IL10	

Yoshida 2005	16172134	HeLa	inserted	IRF1	TRAIL, DCIR	
Yamamoto Plos One 2011	22003407	hematopoietic progenitors		IRF8	macrophage development	
Yamamoto Plos One 2011	22003407	hematopoietic progenitors		IRF4	macrophage differentiation	
Yamamoto Plos One 2011	22003407	hematopoietic progenitors		IRF4	cell cycle arrest	
Yamamoto Plos One 2011	22003407	macrophages		IRF4	phagocytosis	
Vander Lugt Nat Immunol 2014	24362890	DC (mouse)		IRF4	antigen presentation in class II	antigen presentation in class II
Vander Lugt Nat Immunol 2014	24362890	DC (mouse)		IRF4	ZBTB46, CIITA, RELB, H2-DMB2, CTSS	
Vander Lugt Nat Immunol 2014	24362890	DC (mouse)		IRF8	ITGAE (CD103)	
Chopin J Exp Med 2013	24249112	LC (mouse) LC-like (mouse)		PU.1	LC development	
Chopin J Exp Med 2013	24249112	LC (mouse) LC-like (mouse)		IRF4	acquired out of epidermis	
Chopin J Exp Med 2013	24249112	LC (mouse) LC-like (mouse)		IRF4	acquired out of epidermis	
Chopin J Exp Med 2013	24249112	LC (mouse) LC-like (mouse)		PU.1	LC re-population in inflammation	
Chopin J Exp Med 2013	24249112	LC (mouse) LC-like (mouse)		PU.1	RUNX3	LC development
Chopin J Exp Med 2013	24249112	LC (mouse) LC-like (mouse)		RUNX3	LC development	
Giese J Exp Med 1997	9348311	mice		IRF8	Th1	
Liu JBC 2004	15489234	macrophages (mouse)		IRF8 and IRF1	IL12p35	
Kim JI 1999	10438937	macrophage line (mouse)	LPS	IRF8 and PU.1	IL18	
Berghout Plos Pathogens 2013	23853600	brain	neuroinflammation	IRF8	Ccl4, Ccl5, Ccl7, Ccl12, Cxcl9, Cxcl10	chemotaxis
Berghout Plos Pathogens 2013	23853600	brain	neuroinflammation	IRF8	Nlrc5, Ifi205	innate
Berghout Plos Pathogens 2013	23853600	brain	neuroinflammation	IRF8	Oasl2, Mx2, Oas1g	viral infection
Berghout Plos Pathogens 2013	23853600	brain	neuroinflammation	IRF8	Ifit2, Ifit3, Isg15, Rsad2	type1 IFN
Berghout Plos Pathogens 2013	23853600	brain	neuroinflammation	IRF8	C1q, C4b, Fcerg1	antigen capture
Berghout Plos Pathogens 2013	23853600	brain	neuroinflammation	IRF8	Irgm1, Irgm2, Igtp, Gbp2, Gbp3	phagosome maturation
Berghout Plos Pathogens 2013	23853600	brain	neuroinflammation	IRF8	Tap1, Tap2	antigen processing
Berghout Plos Pathogens 2013	23853600	brain	neuroinflammation	IRF8	B2m, H2-Ab1, H2-D, H2-K, H2-L, H2-Q, H2-T22	antigen presentation
Berghout Plos Pathogens 2013	23853600	brain	neuroinflammation	IRF8	Irf1, Irf7, Irf9	early response

Berghout Plos Pathogens 2013	23853600	IRF1-/- mice		IRF1	CD8 Tcell, no effect on CD4	
Akbari JI 2014	24489086	DC (mouse), macrophages (mouse)	L.major infection	IRF4	anti-Th1	
Akbari JI 2014	24489086	DC (mouse), macrophages (mouse)	L.major infection	IRF4	anti-IL12	
Hambleton NEJM 2011	21524210	human IRF-/-	CG infection, immune deficien	IRF8	IL12, partially IFNg, TNFa, IL10	
Hambleton NEJM 2011	21524210	human IRF-/-	CG infection, immune deficien	IRF8	DC differentiation	
Hambleton NEJM 2011	21524210	human IRF-/-	CG infection, immune deficien	IRF8	LC NOT AFFECTED	
Kamijo Science 1994	7510419	macrophages (mouse)		IRF1	NO synthesis	
Marquis Plos Genetics 2011	21731497	macrophages (mouse)	IFNg, CpG, Tuberculosis	IRF8	CD74	
Marquis Plos Genetics 2011	21731497	macrophages (mouse)	IFNg, CpG, Tuberculosis	IRF8	H2-D1	
Marquis Plos Genetics 2011	21731497	macrophages (mouse)	IFNg, CpG, Tuberculosis	IRF8	H2-DMa	
Marquis Plos Genetics 2011	21731497	macrophages (mouse)	IFNg, CpG, Tuberculosis	IRF8	H2-DMb1/2	
Marquis Plos Genetics 2011	21731497	macrophages (mouse)	IFNg, CpG, Tuberculosis	IRF8	H2-Ea	
Marquis Plos Genetics 2011	21731497	macrophages (mouse)	IFNg, CpG, Tuberculosis	IRF8	H2-Eb1	
Marquis Plos Genetics 2011	21731497	macrophages (mouse)	IFNg, CpG, Tuberculosis	IRF8	H2-Q8	
Marquis Plos Genetics 2011	21731497	macrophages (mouse)	IFNg, CpG, Tuberculosis	IRF8	Ltb	
Marquis Plos Genetics 2011	21731497	macrophages (mouse)	IFNg, CpG, Tuberculosis	IRF8	Tapbp1	antigen presentation
Marquis Plos Genetics 2011	21731497	macrophages (mouse)	IFNg, CpG, Tuberculosis	IRF8	Ccl6,	
Marquis Plos Genetics 2011	21731497	macrophages (mouse)	IFNg, CpG, Tuberculosis	IRF8	Cxcl9,	
Marquis Plos Genetics 2011	21731497	macrophages (mouse)	IFNg, CpG, Tuberculosis	IRF8	IL6ra,	
Marquis Plos Genetics 2011	21731497	macrophages (mouse)	IFNg, CpG, Tuberculosis	IRF8	Csfr3,	
Marquis Plos Genetics 2011	21731497	macrophages (mouse)	IFNg, CpG, Tuberculosis	IRF8	Fcgrt,	
Marquis Plos Genetics 2011	21731497	macrophages (mouse)	IFNg, CpG, Tuberculosis	IRF8	Tlr9	chemokines and receptors
Marquis Plos Genetics 2011	21731497	macrophages (mouse)	IFNg, CpG, Tuberculosis	IRF8	Gbp2,3,5,6,	
Marquis Plos Genetics 2011	21731497	macrophages (mouse)	IFNg, CpG, Tuberculosis	IRF8	Gma1,	
Marquis Plos Genetics 2011	21731497	macrophages (mouse)	IFNg, CpG, Tuberculosis	IRF8	Rgl2	anti-microbial GTPases
Marquis Plos Genetics 2011	21731497	macrophages (mouse)	IFNg, CpG, Tuberculosis	IRF8	CTSD	
Marquis Plos Genetics 2011	21731497	macrophages (mouse)	IFNg, CpG, Tuberculosis	IRF8	CTSB	
Marquis Plos Genetics 2011	21731497	macrophages (mouse)	IFNg, CpG, Tuberculosis	IRF8	CTSS	
Marquis Plos Genetics 2011	21731497	macrophages (mouse)	IFNg, CpG, Tuberculosis	IRF8	SLC15A3	
Marquis Plos Genetics 2011	21731497	macrophages (mouse)	IFNg, CpG, Tuberculosis	IRF8	CD74	endolytic pathway class II presentation
Marquis Plos Genetics 2011	21731497	macrophages (mouse)	IFNg, CpG, Tuberculosis	IRF8	Ifitm1	early response pathways in myeloid cells
Becker Blood 2012	22238324	myeloid and lymphoid progenitors		IRF8		DC lineage
Schlitzer Immunity 2012	23706669	DC (mouse, human)		IRF4	IL23	Th17
Persson Immunity 2013	23664832	DC (mouse, intestinal)		IRF4		Th17
Yan Virology 2004	15207617	MoLCs and MoDCs		IRF/ETS	Caveolin	Antigen cross-presentation
Antonios JI 2010	20525893	MoDCs	NiSO4	IRF	IL12p70	

Table S5. Genes regulated by expression programme "A" and "B" in the IRF-GRN

Gene Symbol	in silico TNFa/TSLP	expression values: microarray average max TNFa -T0/ average TSLP-T0
HLA-F	Programme "A"	1.661487945
CAV1	Programme "A"	1.445900654
CD40	Programme "A"	1.30052486
CD80	Programme "A"	1.236425487
TAPBPL	Programme "A"	1.233496426
CYBB	Programme "A"	1.181947045
HLA-A	Programme "A"	1.16959572
HLA-E	Programme "A"	1.164051773
CD86	Programme "A"	1.161503657
HLA-C	Programme "A"	1.160652509
IL15	Programme "A"	1.153998136
ERAP2	Programme "A"	1.149261298
NOS2	Programme "A"	1.116805394
PSME2	Programme "A"	1.110154397
TAP1	Programme "A"	1.08352888
ERAP2	Programme "A"	1.082775362
PSMB8	Programme "A"	1.041911729
HLA-B	Programme "A"	1.03594324
TAP2	Programme "A"	1.035112542
PSMB10	Programme "A"	1.025640822
IFNB	Programme "A"	1.019397771
TAPBP	Programme "A"	1.003928019
IL18BP	Programme "A"	1.000227808
PSME1	Programme "A"	0.966082654
TAP1	Programme "A"	0.963070421
ERAP1	Programme "A"	0.863455796
B2M	Programme "A"	0.806266221
IL18	Programme "A"	0.702631966
LYZ	Programme "B"	0.714557952

HLA-DPB1	Programme "B"	0.719979728
HLA-DQB1	Programme "B"	0.750181119
PRDM1	Programme "B"	0.791913837
CTSL1	Programme "B"	0.7944213
CTSD	Programme "B"	0.812923669
CTSB	Programme "B"	0.828107658
IL33	Programme "B"	0.879277268
CTSC	Programme "B"	0.881195132
CTSS	Programme "B"	0.910063224
CTSL2	Programme "B"	0.910461669
CIITA	Programme "B"	0.94038742
SLC15A3	Programme "B"	1.004945473
CST3	Programme "B"	1.021201608
CD74	Programme "B"	1.043747899
HLA-DMB	Programme "B"	1.047604139
HLA-DOB	Programme "B"	1.062740136
PSAP	Programme "B"	1.06518009
HLA-DRB1	Programme "B"	1.089017639
HLA-DQA1	Programme "B"	1.093025879
CTSL3	Programme "B"	1.20392131
IL10	Programme "B"	1.291658339

Table S6. Experimentally measured expression values at 0h (0-8 time block),2h (9-32 time block),8h (33-75 time block),and 24h (76-100 time block) converted to parametrisation values for each GRN entry node

	LC TNFa	LC TSLP
**IRF1**	0-8,325;9-32,1267;33-75,1209;76-100,1782	0-8,293;9-32,841;33-75,585;76-100,796
**IRF8**	0-8,89;9-32,879;33-75,200;76-100,131	0-8,63;9-32,847;33-75,203;76-100,206
**IRF4**	0-8,3762;9-32,4296;33-75,3067;76-100,2961	0-8,3773;9-32,4618;33-75,3638;76-100,5034
**cJUN**	0-8,2206;9-32,4831;33-75,3571;76-100,2797	0-8,2204;9-32,4798;33-75,3147;76-100,2207
**cFOS**	0-8,1072;9-32,811;33-75,153;76-100,34	0-8,1125;9-32,783;33-75,109;76-100,43
**BATF**	0-8,259;9-32,490;33-75,393;76-100,513	0-8,259;9-32,449;33-75,290;76-100,276
**BATF3**	0-8,174;9-32,299;33-75,511;76-100,697	0-8,174;9-32,270;33-75,325;76-100,469
**ELF1**	0-8,650;9-32,1112;33-75,724;76-100,521	0-8,669;9-32,1234;33-75,692;76-100,457
**ELF4**	0-8,159;9-32,244;33-75,204;76-100,198	0-8,155;9-32,238;33-75,181;76-100,163
**ELK1**	0-8,182;9-32,172;33-75,200;76-100,175	0-8,170;9-32,182;33-75,168;76-100,176
**ELK3**	0-8,194;9-32,273;33-75,249;76-100,261	0-8,272;9-32,317;33-75,248;76-100,423
**ETS1**	0-8,775;9-32,868;33-75,883;76-100,972	0-8,849;9-32,935;33-75,900;76-100,1292
**ETS2**	0-8,404;9-32,413;33-75,225;76-100,118	0-8,389;9-32,463;33-75,250;76-100,130
**EHF**	0-8,92;9-32,133;33-75,117;76-100,295	0-8,105;9-32,146;33-75,112;76-100,229
**ELF2**	0-8,234;9-32,306;33-75,209;76-100,252	0-8,241;9-32,341;33-75,231;76-100,243
**ETV3**	0-8,956;9-32,889;33-75,544;76-100,785	0-8,884;9-32,843;33-75,563;76-100,749
**ETV6**	0-8,558;9-32,412;33-75,363;76-100,392	0-8,527;9-32,489;33-75,438;76-100,448
**GABPa**	0-8,141;9-32,121;33-75,167;76-100,184	0-8,144;9-32,190;33-75,243;76-100,234

Table S7. Experimentally measured expression values for input nodes in LC migrating in the presence or absence of PI3K-gamma inhibitor, AS605240, average of n=2 independent donors

	medium	AS605240
**IRF1**	132.7546817	95.80075732
**IRF8**	62.16377096	49.23235916
**IRF4**	1100.954543	1018.206481
**cJUN**	337.125426	289.1024671
**cFOS**	39.07982017	37.31223789
**BATF**	18.8818089	20.11738465
**BATF3**	40.12633954	41.21470658
**ELF1**	1042.382563	1075.905004
**ELF4**	136.0850999	89.37748514
**ELK1**	123.4924816	91.81755367
**ELK3**	48.35778858	42.77809158
**ETS1**	157.9167944	127.3214019
**ETS2**	162.6078164	133.8398353
**EHF**	64.18514444	62.96805486
**ELF2**	53.8216346	48.73587259
**ETV3**	704.7474309	571.9613531
**ETV6**	483.8379809	437.0045973
**GABPa**	100.114365	103.6889617
